# PSG6: A mitochondrially-targeted gentisic acid derivative exerts antiplatelet action via mitochondrial complex I inhibition

**DOI:** 10.1016/j.redox.2026.104200

**Published:** 2026-05-06

**Authors:** Francisca Tellería, Matías Monroy-Cárdenas, Cristina Pecorilla, Amina Djurabekova, Diego Méndez, Magdalena Sepúlveda, Felipe Lagos, Santiago Mansilla, Laura Castro, Andrés Trostchansky, Adriana Covarrubias-Pinto, Alexis González, Ivan Dikic, Iván Palomo, Volker Zickermann, Vivek Sharma, Lisandra Morales-Malvarez, Héctor Montecino-Garrido, Ramiro Araya-Maturana, Eduardo Fuentes

**Affiliations:** a Thrombosis and Healthy Aging Research Center, VITALIS Longevity Center, Medical Technology School, Department of Clinical Biochemistry and Immunohematology, Faculty of Health Sciences, Universidad de Talca, Talca, Chile; b Instituto de Química de Recursos Naturales, Universidad de Talca, Talca, Chile; c Department of Physics, University of Helsinki, Helsinki, Finland; d Departamento de Métodos Cuantitativos and Centro de Investigaciones Biomédicas (CEINBIO), Facultad de Medicina, Universidad de la República, Montevideo, 11800, Uruguay; e Departamento de Bioquímica and Centro de Investigaciones Biomédicas (CEINBIO), Facultad de Medicina, Universidad de la República, Montevideo, 11800, Uruguay; f Institute of Biochemistry II, University Hospital, Goethe University, Frankfurt am Main, Germany; g HiLIFE Institute of Biotechnology, University of Helsinki, Helsinki, Finland

**Keywords:** Antiplatelet, Triphenylphosphonium, Gentisic acid, Mitochondria, complex I

## Abstract

Cardiovascular diseases are the leading cause of death worldwide, and cancer-associated thrombosis remains a major clinical challenge because of the interplay between tumour progression and platelet activation. Platelets contribute to thrombus formation by adhering to damaged endothelium and undergoing aggregation. Since mitochondria-targeted compounds are useful as antitumour and antiplatelet agents, we evaluated a series of triphenylphosphonium salts derived from gentisic acid alkyl esters with varying chain lengths, searching for antiplatelet agents with dual activity. The compound with a six-carbon chain (PSG6) exhibited the highest antiplatelet activity without increasing bleeding risk, whereas the cytotoxicity was found to increase with the chain length. PSG6 also showed selective anticancer effects, reducing tumour cell viability at micromolar concentrations, inducing mitochondrial fission, and lowering the mitochondrial membrane potential (ΔΨm) at 10 μM. Mechanistically, PSG6 decreased ΔΨm, inhibited the mitochondrial electron transport chain (ETC) at complex I, and increased intracellular calcium and reactive oxygen species (ROS) production. Complex I inhibition was confirmed in the yeast model organism *Yarrowia lipolytica* (IC_50_ = 2.9 μM), and atomistic molecular dynamics simulations suggest that PSG6 may inhibit complex I by binding at the shallow site of the ∼30 Å long ubiquinone tunnel. These results position PSG6 as a promising mito-inhibitor candidate for antiplatelet therapy.

## 1 Introduction

Mitochondria are essential dynamic organelles that manage some key cellular functions, including adenosine triphosphate (ATP) production, metabolism regulation, and various signalling pathways [1,2]. Mitochondrial oxidative phosphorylation (OXPHOS) links nutrient oxidation to ATP synthesis through a sequence of redox reactions in the electron transport chain (ETC). However, the metabolic roles of mitochondria extend well beyond bioenergetics; they also create biosynthetic precursors for macromolecules and organise metabolites to maintain redox balance. Central to these functions is the activity of the ETC, which reduces molecular oxygen to water at complex IV [3] and couples this reaction to proton translocation from the mitochondrial matrix to the inter-membrane space, establishing both the proton gradient and the mitochondrial membrane potential, together forming the proton motive force [4,5]. This allows the ATP synthase enzyme (complex V) to generate ATP using the movement of protons going into the matrix [1]. Because of mitochondria's role in various cellular functions, it serves as a significant pharmacological target in some main disease conditions.

Although hydroquinones have shown great potential to impact ETC, the pharmacological use of most of them is limited because they cannot adequately reach the mitochondrial inner membrane [6]. To address this, several mitochondrial-targeted drugs have been developed, using mainly cancer cell models [7]. Among these, lipophilic cation-based compounds like ubiquinone derivatives are an innovative approach because they can accumulate selectively in mitochondria by taking advantage of the organelle's negative membrane potential [[8], [9], [10]].

Linking hydroquinone to the lipophilic triphenylphosphonium (TPP) cation enhances both membrane permeability and mitochondrial accumulation [11,12]. The length of the alkyl linker between TPP and the hydroquinone moiety influences the compound's hydrophobicity, membrane penetration, and cytotoxicity [13]. We have previously shown that this class of compounds can disrupt mitochondrial function without inducing toxicity, thereby impairing mitochondrial energy production, a process essential for platelet activation [[14], [15], [16]].

Platelets are essential, enucleated cells that play a critical role in haemostasis. Platelet activation relies on pathways that require extensive cytoskeletal reorganisation, an energy-demanding process that depends heavily on ATP production [[17], [18], [19], [20]], calcium signalling [18,21], and redox regulation through reactive oxygen species (ROS) [22], all of which are tightly controlled in mitochondrial functioning [23]. Given their strong dependence on mitochondrial activity, mitochondria-targeted compounds have the potential to disrupt platelet adaptive responses and interfere with their pathological metabolic processes [22,[24], [25], [26]]. This study aims to evaluate TPP salts derived from gentisic acid alkyl esters, focusing on how their linker length influences key pharmacological and biophysical parameters in the search for new antiplatelet agents.

## 2 Methods

### 2.1 Chemical synthesis

#### 2.1.1 General methods

**Scheme 1:**
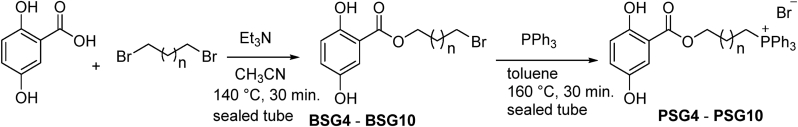
Synthesis of phosphonium-salts of gentisic acid alkyl esters.

**Fig. 1:**
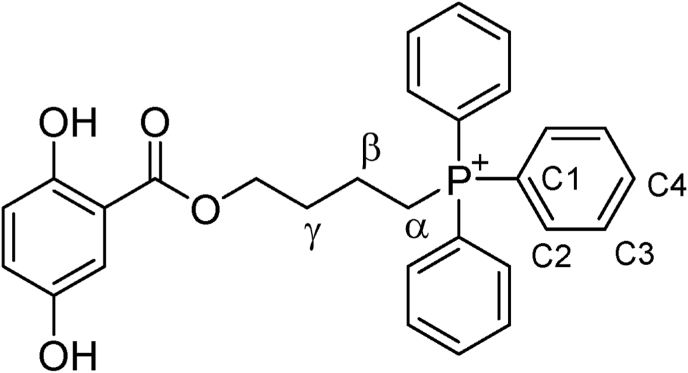
Labels used to assign couplings.

The synthetic method of the studied compounds (Table 1) is depicted in Scheme 1 and Supplementary Methods. ^1^H and ^13^C NMR spectra were obtained using a spectrometer operating at either 400.13 MHz (^1^H) or 100.61 MHz (^13^C), with CDCl_3_ as the solvent. Chemical shifts are reported as ppm downfield from TMS for ^1^H NMR and relative to the central CDCl_3_ resonance (77.0 ppm) for ^13^C NMR. High-resolution mass spectra (HRMS) were obtained using a Bruker Compact Q-TOF MS (ESI/QTOF). Silica gel 60 (230–400 mesh ASTM) and TLC sheets silica gel 60 F254 were used for flash-column chromatography and analytical TLC, respectively. ^31^P couplings with ^1^H and ^13^C were assigned by comparison with the reported *J* values [27,28]. The numbering and labels of atoms in the phosphonium salts are shown in Fig. 1.

**Table 1:** Studied compounds.

Compound	PSG4	PSG5	PSG6	PSG8	PSG10
n	2	3	4	6	8

### 2.2 Biological experiments

#### 2.2.1 Blood collection

Venous phlebotomy was performed on voluntary donors (male and female between 18 and 40 year old, apparently healthy –no chronic or known conditions–, and 10 days without AINEs medication) who agreed to participate in the study through informed consent (protocol approved by the Scientific Ethics Committee of the University of Talca, No. 04-2022) [29]. For the extraction, acid citrate dextrose (ACD) was mixed with the whole blood in a ratio of 4:1 *v/v*.

#### 2.2.2 Purification of washed human platelets

Whole blood was centrifuged for 10 min at 200 *g* to obtain platelet-rich plasma (PRP). The PRP obtained was supplemented with PGI2 (1 μg/mL) and centrifuged for 10 min at 100 *g* to remove other cells. The supernatant (PRP) was then transferred to a new tube and centrifuged for 10 min at 900 g. The platelet pellet was gently re-suspended in 5 mL of Tyrode's buffer without calcium plus ACD at 9:1 *v*/*v* and centrifuged for 10 min at 900 *g*. Finally, the platelet pellet was resuspended in a Tyrode's buffer without calcium. All centrifugations were done at room temperature (RT), with acceleration = 6 and without brake. The final concentration of platelets for each experiment was adjusted with the Mindray BC-3000 Plus haematology counter, Japan [15,30].

#### 2.2.3 Cytotoxic activity

Washed platelets (200–250 × 10^6^ platelets/mL) were incubated with the compound (1-40 μM) for 10 min at 37 °C. DMSO 0.2% was used as the vehicle. Platelets were then centrifuged at 900 *g* for 10 min to obtain the supernatant, which was mixed with the working reagent of the Lactate Dehydrogenase (LDH) Cytotoxicity Kit (Cayman Chemical, Ann Arbor, MI, USA). The maximum cytotoxicity control corresponds to Triton X-100 at 10% [15,31].

#### 2.2.4 Flow cytometry analysis

The platelet population (>99 %) in whole blood, platelet-rich plasma and washed platelets was detected using anti-Human CD61 FITC (BD Biosciences, San Jose, CA, USA Item 348093) (Supplementary Fig. 1) [32]. Data was extracted as mean fluorescence intensity (MFI) or percentage of positivity in platelet population.

#### 2.2.5 Cell viability

Washed platelets (200–250 × 10^6^ platelets/mL) were labelled with Calcein-AM and incubated for 20 min at 37 °C in the dark. Subsequently, the compound (1-40 μM) was added and incubated for 10 min at 37 °C in the dark. DMSO was used as the vehicle. Next, the population of CD61-positive platelets was identified, and their viability was determined with the BD FacsLyric flow cytometer (BD Biosciences, San Diego, CA, USA). The percentage of calcein-negative platelets in the CD61-positive subpopulation was recognised as non-viable. Triton X-100 0.1% was used as the cell damage control [15,26].

#### 2.2.6 Apoptosis activity (externalization of phosphatidylserine)

Washed platelets (200–250 × 10^6^ platelets/mL) were incubated with the compound (1-40 μM) for 10 min at 37 °C. DMSO was used as the vehicle. An aliquot from each condition was incubated with Annexin-V FITC to identify increased externalization of phosphatidylserine (PS) with the BD Facs Lyric flow cytometer (Annexin V-FITC Apoptosis Detection/Staining Kit, ABCAM, Boston, MA, USA). The apoptotic platelet population was identified as the fraction (%) of Annexin V positive platelets. As a positive control of procoagulant/apoptosis activity, hyperactivated platelets were used with a mixture of collagen (2 μg/mL) and TRAP-6 (10 μM) [15,26].

#### 2.2.7 Platelet aggregation

Platelet aggregation was evaluated in washed platelets (200–250 × 10^6^ platelets/mL) in an AggRAM Analyzer aggregometer (Helena Laboratories, Beaumont, TX, USA). Washed platelets were preincubated with the compound at different concentrations for 5 min at 37 °C inside the aggregometer. Then, aggregation was initiated with TRAP-6 (5 μM) or collagen (2 μg/mL). The aggregation reaction was measured for 5 min at 37 °C under continuous stirring (1000 rpm) [15,33].

#### 2.2.8 Platelet activation markers

Washed platelets (200–250 × 10^6^ platelets/mL) were preincubated with the compound (5 and 10 μM) or vehicle (DMSO 0.2%) for 5 min at 37 °C and then activated with 5 μM TRAP-6 or 2 μg/mL collagen followed by 5 min of incubation at 37 °C. Subsequently, aliquots were taken and labelled separately with each of the antibodies against the activation markers P-selectin (CD62p), and Fibrinogen binding (anti-fibrinogen). The reading was performed on a BD FacsLyric flow cytometer (BD Biosciences, San José, CA, USA). CD61 FITC was used to identify the platelet population [15,31]. The same protocol described was used for activation controls in whole blood and PRP (Supplementary Fig. 2).

#### 2.2.9 Clot retraction

Blood was obtained in 3.2% citrate tubes and centrifuged for 12 min at 250 g to obtain platelet-rich plasma (PRP). The assay was prepared in Khan tubes by adding 750 μL of Tyrode's buffer without calcium, 200 μL of PRP, and 5 μL of red blood cells; the mixture was treated with compound at 1 and 10 μM or Eptifibatide at 10 μM as inhibitory control and incubated for 10 min. A glass rod was positioned in the center of the tube (to support the clot adhesion) and clot retraction was initiated by adding 50 μl of 10 Units/mL thrombin. Photographs and the clots formed after 2 h were recorded and weighed to quantify retraction [34].

#### 2.2.10 Bleeding time *in vitro*

Blood was obtained in 3.2% citrate tubes, which were allowed to settle for 30 min before the test. Whole blood was mixed with the compound at 1 and 10 μM and incubated for 10 min. Subsequently, the sample was loaded into the Collagen/Epinephrine or Collagen/ADP cartridge, and the closure time was measured in the Innovance PFA-200 system (Siemens Healthcare Diagnostics Products, Munich, Germany). Eptifibatide 10 μM was used as a control [35].

#### 2.2.11 *In vitro* thrombosis assay

This assay was adapted from the protocol reported by Rodriguez et al. [36], and performed using Vena8 microfluidic chips (Cellix, Dublin, Ireland). Briefly, whole blood anticoagulated with 3.2% sodium citrate was labelled with DIOC-6 (25 nM) and incubated for 1 h at 37 °C. In parallel, the channels of the Vena8 BioChip were coated with collagen (1 mg/mL) and maintained for 1 h at room temperature in a humidified environment. Before perfusion, the labelled blood was treated with either vehicle or the compound PSG6 (10 μM) for 5 min at 30 °C. Thrombus formation was initiated by perfusing the samples through the collagen-coated channels under a shear rate of 20 dyn/cm^2^ [37]. Images were acquired using a Nikon Ti2 confocal fluorescence microscope with a 40× objective. Quantitative analysis was performed using ImageJ software (version 1.26t, NIH).

#### 2.2.12 Mitochondrial membrane potential (ΔΨm)

Washed platelets (50 × 10^6^ platelets/mL) were labelled with the 100 nM tetramethylrhodamine methyl ester perchlorate (TMRM) potentiometric probe and incubated for 20 min at 37 °C in the dark. Then, the different concentrations of the compound (1 to 100 μM) were added and incubated for 20 min at 37 °C in the dark. The reading was performed on a BD FacsLyric flow cytometer (BD Biosciences, San José, CA, USA). The vehicle (basal control) was DMSO at 0.2%, while the mitochondrial depolarization control was carbonyl cyanide-*p*-trifluoromethoxyphenylhydrazone (FCCP) 1 μM [15,30].

#### 2.2.13 Intraplatelet ROS levels

Reactive oxygen species (ROS) levels were determined in washed platelets (5 × 10^6^ platelets/mL) using dihydroethidium (DHE, 10 μM). The platelets were incubated with DHE for 20 min at 37 °C in the dark, followed by another 10 min incubation with different compound concentrations (1 to 100 μM). The reading was performed on the BD FacsLyric flow cytometer (BD Biosciences, San José, CA, USA). DMSO at 0.2% was used as a basal control, while Antimycin A at 10 μM was used as a positive control for ROS increase [15,38].

#### 2.2.14 Intraplatelet calcium levels

Washed platelets (50 × 10^6^ platelets/mL) were labelled with Fluo-4-AM (0.44 μM) and incubated for 30 min at room temperature in the dark. Next, the different concentrations of the compound (1 to 100 μM) were incubated for 10 min at 37 °C. The reading was performed on a BD FacsLyric Flow Cytometer (BD Biosciences, San José, CA, USA). DMSO at 0.2% was used as a negative control, and carbonyl cyanide-*p*-trifluoromethoxyphenylhydrazone (FCCP) 1 μM was used as a positive control for increased intracellular calcium [15,38].

#### 2.2.15 Oxygen consumption and extracellular acidification rate

Oxygen consumption rate (OCR) was measured with a Seahorse XFe24 extracellular flux analyser (Agilent, Santa Clara, CA, USA). Briefly, 100 μL of platelets washed in modified Tyrode-HEPES buffer were seeded (20–25 × 10^6^ cells/well) and centrifuged at 300 g for 10 min without brake. Platelets were incubated with the compound at 10 μM in a Tyrode's-HEPES buffer, which was then removed and replaced with 500 μL of Seahorse medium (8.3 g/L DMEM, 1.85 g/L NaCl, 5 mM glucose, 1 mM pyruvate, 2 mM glutamine, 5 mM HEPES, pH 7.4) [15]. OCR was measured before and after the sequential addition of 3 μg/mL collagen or seahorse medium, 2.5 μM oligomycin, 1.4 μM FCCP, and 2 μM/2 μM antimycin A/rotenone. Non-mitochondrial OCR was subtracted from all measurements. Respiratory parameters obtained with Mito Stress Test (Supplementary Table 1) were calculated as follows: Baseline (baseline OCR), collagen (OCR after collagen addition), activation (collagen-basal), ATP-independent or proton leak (OCR resistant to oligomycin addition), ATP-linked respiration (basal-proton leak), maximum (OCR obtained after addition of FCCP), and spare capacity (maximal-basal). Respiratory parameters were calculated according to Refs. [39,40].

#### 2.2.16 Immunofluorescence

U2-OS osteosarcoma cells (ATCC, HTB-96) were cultured in standard DMEM medium (Gibco) supplemented with 10% fetal bovine serum (Gibco) and 100 μg/ml penicillin/streptomycin (Thermo Fisher Scientific) and maintained at 37 °C with 5% CO_2_. For immunofluorescence, cells were seeded on black, clear flat-bottom 24-well plates. Following treatment (0.2% DMSO or compound at 5 μM and 20 μM), cells were washed twice with PBS and fixed with 4% paraformaldehyde in PBS for 10 min. After fixation, cells were permeabilized with 0.2% (v/v) Triton X-100 in PBS for 10 min and blocked with 5% BSA in PBS for 1 h. For automated quantitative analysis, images were obtained on the Yokogawa CQ1 confocal quantitative image cytometer (63× magnification). Image analysis was performed using the CQ1 Yokogawa CellPathfinder high-content analysis software. Representative images from at least three independent experiments were collected [41].

#### 2.2.17 Study of complex I

Mitochondrial membranes from *Yarrowia lipolytica* cells were prepared as previously described by Angerer et al. [42]. Specific NADH:hexaammineruthenium (HAR) and deamino-NADH:decylubiquinone (DBQ) oxidoreductase activity was measured for mitochondrial membrane, as described below.

#### 2.2.18 Determination of catalytic activity

NADH:HAR activity of purified complex I was measured at 30 °C in a buffer containing 20 mM Na+/HEPES pH 8.0, 250 mM sucrose, 2 mM EDTA and 2 mM NaN3 using 2 mM HAR and 200 μM NADH as substrates. To determine NADH:ubiquinone oxidoreductase activity, 60 μM DBQ and 100 μM NADH were used as substrates. NADH oxidation rates were recorded with a Shimadzu Multi Spec-1501 diode array spectrophotometer (ε340–400 nm = 6.1 mM−1 cm−1). To analyse the effect of the compounds, aliquots at different concentrations were added to the buffer before starting the reaction with DBQ [43].

#### 2.2.19 Docking of triphenylphosphonium salts of gentisic acid alkyl esters

The cryo EM structure of inhibitor-bound complex I (PDB ID: 7B93), with a resolution of ∼3.04 Å was downloaded from the Protein Data Bank (PDB) [44]. The structure was truncated with the same selection made by Haapanen et al. [45] (ND3, ND1, 49 kDa, 30 kDa, PSST, TYKY) to maintain the minimum scaffold for the CoQ site. The structure was prepared using the Schrödinger's protein preparation wizard module [46,47]. Missing sidechains and hydrogens atoms were added, bond orders were assigned, protonation states were described according to the pH 8, and side chain amides were fixed, while the water molecules and other ligands were removed. The resulting structure was energy minimized. The PSG6 molecule was prepared with the ligand preparation module. The docking process was carried out using the Glide XP software [48], with a grid box centred on the position of the inhibitor bound in the CoQ tunnel of complex I (PDB ID:7B93). The top 10 docking poses were selected as defined by free energy calculations using the MM-GBSA software [49].

#### 2.2.20 Molecular dynamics simulations

Classical all-atom molecular dynamics (MD) simulations were performed using the high-resolution cryo-EM structure of complex I from *Yarrowia lipolytica* (PDB ID: 7O71) [50]. We chose the yeast structure because activity assays of CoQ reduction in the presence of PSG6 were performed on the complex from the same species (see above). In addition, this is also one of the highest-resolution cryo-EM structures available in the PDB (local resolution up to ∼2.1 Å) and shows several structural features that are commensurate with the deactive- (open-like) states of complex I. Notably, deactive-(open-like) states are known to be present in higher proportions in several complex I preparations [50,51]. To achieve long time scale dynamics, we restricted the size of the model system, by excluding the protein subunits that are distant from the CoQ binding site (see Supplementary Table 2). Similar, truncated complex I model systems have successfully been used in the past to obtain functional insights (see e.g. Refs. [[52], [53], [54]]). Non-standard protonation states of titratable amino acid residues were obtained with PropKa [55] (see Supplementary Table 2). The coordinates of PSG6 were obtained from the docking poses (see above) and parameterized using the CHARMM-GUI Ligand Reader and Modeler [56,57] (see Supplementary Data and Supplementary Table 4). To enable direct comparison between PSG6 and a native substrate, we selected coenzyme Q9 (CoQ9) as a reference molecule. CoQ9 binds in the ∼30 Å long CoQ binding cavity of complex I at several locations within the tunnel [45,58,59]. The CoQ binding sites 1-2 (deep binding sites) are closer to the N2 FeS cluster, whereas sites 4-5 (shallow binding sites) are closer to the tunnel entrance. Oxidized CoQ9 force field parameters were taken from earlier work [60].

In the simulation systems, the PSG6 binding pose was modeled based on the docking conformations obtained from Glide (see above). The CoQ9 was aligned to the same site, which corresponds to the site 4/5 region of the CoQ tunnel, and where CoQ molecules have been resolved in earlier cryo-EM studies. Both ligand-bound protein complexes were embedded in a lipid bilayer composed of 3-palmitoyl-2-oleoyl-d-*glycero*-1-Phosphatidylcholine (POPC), 3-palmitoyl-2-oleoyl-d-*glycero*-1-Phosphatidylethanolamine (POPE), and tetralinoleoyl Cardiolipin (TLCL), in a ratio of 10:7:3 ratio, corresponding to the inner mitochondrial membrane composition. Thereafter, the protein-membrane model was solvated in TIP3P water solvent. The protein, membrane and water system was electrically neutralized (by 120 Na^+^ ions, see Supplementary Table 4), followed by the addition of 0.10 M Na^+^/Cl^−^ [[61], [62], [63], [64]].

Initial energy minimization was conducted in two stages. First, using NAMD [65], the heavy atoms of the protein, lipid phosphorus atoms, and ligands head groups were harmonically restrained (force constant k ≈ 41,800 kJ mol^−1^ nm^−2^); second, a short minimization was performed without restraints with GROMACS 2021.6 [66]. The first equilibration step was conducted in NVT ensemble with restraints (20000 kJ mol^−1^ nm^−2^) on protein, ligands, and the phosphorus atoms of the lipids along the membrane normal direction until system reached a temperature of 310K, using V-rescale thermostat [67], for a total of 100 ps. This was followed by a series of NPT restrained equilibrations, each lasting 10 ns, where the harmonic restraints on protein heavy atoms progressively decreased stepwise from 20000 to 12800, then 7200, 3200, and finally 800 kJ mol^−1^ nm^−2^ while restraints (2000 kJ mol^−1^ nm^−2^) were kept on heavy atoms of compound head groups. A final NPT equilibration was then performed for 20 ns, with no restraints applied. During this phase, one frame was saved every 2.5 ns (five total) from the second half of this equilibration step. Each saved frame was subjected to an additional 10 ns NPT equilibration after generation of a new random seed, followed by 500 ns of production in order to generate five independent replicas for both CoQ9 and PSG6.

Temperature and pressure were controlled using the V-rescale thermostat (310 K) and Berendsen barostat [68] (1 bar) during equilibration. Production simulations used the Nose–Hoover thermostat [69,70] and Parrinello–Rahman barostat [71]. The timestep was 2 fs via the LINCS algorithm [72]. Electrostatics were treated using the particle-mesh Ewald method [73] with a 12 Å cutoff. The van der Waals interactions cutoff was 12 Å, with a switching distance at 10 Å. During production, a frame every 100 ps was saved for later analyses.

Steered MD (SMD) simulations were carried out for both PSG6 and CoQ9. Five independent SMD simulations per ligand (each initiated from the last frame of the 500 ns production run) were performed using GROMACS *pull* code. Ligands were pulled along a defined vector, from the ligand head towards Tyr144 of the 49 kDa subunit, at a constant velocity of 1 Å/ns, using a force constant of 5000 kJ mol^−1^ nm^−2^. The choice of the pulling direction was made in order to pull both compounds along a relative straight line, towards Q binding site 1 direction. Pulling force was saved every 0.1 ps for later analyses.

To probe the stability and dynamics of PSG6 in protein-free membrane, model systems comprising PSG6 molecule in a lipid bilayer corresponding to the inner mitochondrial membrane composition (see above) were also simulated (see Supplementary Table 4). The dynamics of improper dihedral angle of PSG6 was analysed from MD simulations corresponding to PSG6-bound to complex I and PSG6 in membrane (see Fig. 7 and Supplementary Fig. 14).

Data from simulations was processed and visualized using VMD [74]. Additional data analysis was performed using GROMACS, VMD, and Jupyter [75]. The simulation related files are available for download from Zenodo server (https://doi.org/10.5281/zenodo.19627373).

***Statistical analysis.*** Data were analysed with GraphPad Prism 10.5.0 (673) software (GraphPad Inc., San Diego, CA, USA) and expressed as the mean ± standard error of the mean (SEM). Each *n* represents an independent biological replicate, defined as platelets obtained from distinct donors. Sample sizes were consistent within individual experiments, and each dataset included in a given analysis was internally uniform. Differences between groups were analysed using a one-way analysis of variance (ANOVA) and Bonferroni's post hoc test unless stated otherwise. *p* values < 0.05 were considered statistically significant.

## 3 Results

### 3.1 PSG6 exerts potent antiplatelet activity while preserving cell viability and haemostasis

**Fig. 2:**
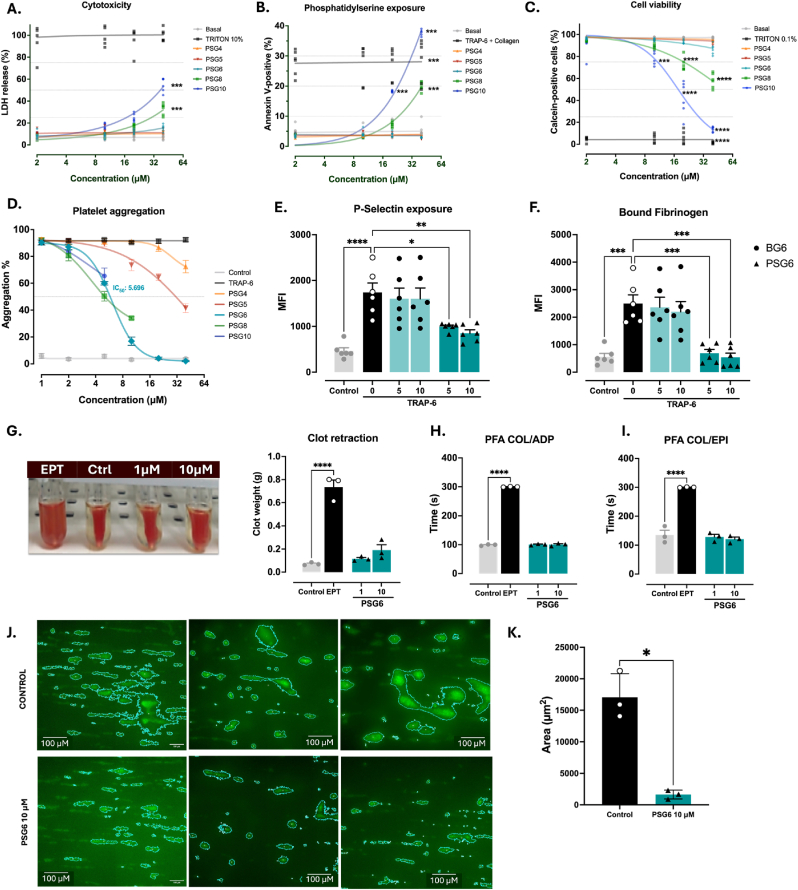
**PSG6 inhibits platelet function without inducing cytotoxicity or impairing hemostasis**. (A) Platelet cytotoxicity by LDH release. (B) Platelet apoptosis by Annexin-V (phosphatidylserine externalization). (C) Platelet viability by Calcein-AM. (D) Platelet aggregation induced with TRAP-6 at 5 μM of gentisic acid linked to TPP by different chain lengths. (E) P-Selectin expression (PE-CD62p). (F) Activated IIb/IIIa (FITC-Fibrinogen) (G) Representative image of clot retraction assay and clot weight after 2 h. (H–I) PFA-200 closure times in collagen/ADP and collagen/epinephrine cartridges, respectively. Eptifibatide (EPT) 10 μM was used as a control for G–I. (J) Representative fluorescence microscopy images of thrombus formation on 3 different areas of collagen-coated channels under arterial shear conditions, showing vehicle (DMSO 0.2%) versus PSG6 at 10 μM. Thrombus coverage is expressed as the percentage of surface area occupied by fluorescently marked platelets. (K) Quantification of thrombus area based on fluorescence intensity of platelet-specific markers, using a defined threshold to exclude non-platelet signals. For A-F, data is shown as mean ± SEM (n = 6). One-way ANOVA – Bonferroni was performed as a statistical analysis. ∗p < 0.05; ∗∗p < 0.01; ∗∗∗p < 0.001; ∗∗∗∗p < 0.0001 vs vehicle (DMSO 0.2%). For G-K, data are -presented as mean ± SEM (n = 3). One-way ANOVA with Bonferroni's post hoc test was used for statistical analysis. ∗p < 0.05, ∗∗p < 0.01, ∗∗∗p < 0.001 vs. vehicle (DMSO 0.2%).

The cytotoxicity of the synthesized compounds was evaluated in platelets at concentrations ranging from 1 to 40 μM (Fig. 2A–C). Compounds bearing alkyl chain linkers of six carbons or fewer did not show cytotoxic effects at any tested concentration. In contrast, starting at 10 μM, PSG8 and PSG10 induced a significant increase in LDH release (Fig. 2A), enhanced apoptosis as indicated by phosphatidylserine externalization (Fig. 2B and Supplementary Fig. 3), and reduced platelet viability (Fig. 2C and Supplementary Fig. 4). Interestingly, when we evaluated the effects of PSG6 (0.5–50 μM) on different breast cell lines—tumourigenic (MD-MBA-231 and MCF7) and non-tumourigenic (MCF10F)—we found a marked reduction in relative viability for both tumourigenic lines starting at 1 μM. In contrast, the viability of MCF10F remained largely unaffected at concentrations below 25 μM (Supplementary Fig. 5). Overall, cytotoxicity correlated positively with both alkyl chain length and compound concentration.

The antiplatelet activity of the synthesized compounds was subsequently evaluated by aggregometry and by measuring P-selectin exposure and fibrinogen binding –standard markers of platelet activation– using different agonist. As shown in Fig. 2D, only PSG6 produced significant inhibition of platelet aggregation within the non-cytotoxic range, with an IC_50_ of 5.7 ± 0.5 μM for TRAP-6. When evaluated with collagen and thrombin, PSG6 showed an IC_50_ of 6.02 ± 1.19 μM and over 20 μM when stimulated with collagen and thrombin, respectively (Supplementary Fig. 6). Given its capacity to reduce platelet aggregation, PSG6 was selected for subsequent experiments, along with its control compound BSG6, a non-TPP^+^ analogue with the same alkyl chain length and gentisic acid head group. Platelets incubated with PSG6 at 5 and 10 μM exhibited a significant, dose-dependent reduction in P-selectin expression and bound fibrinogen levels, whereas BSG6 had no effect on either marker (Fig. 2E and F, Supplementary Figs. 7 and 8).

Since PSG6 markedly attenuate platelet aggregation and activation, we next evaluated whether its antiplatelet effects translated into reduced thrombus formation and potential bleeding risk *in vitro*. To this end, we performed clot retraction assays, PFA-200 closure time measurements using epinephrine/collagen and ADP/collagen cartridges, and thrombosis assays under flow conditions. As shown in Fig. 2G–I, PSG6 did not affect clot retraction (Fig. 2G) or closure times (Fig. 2H and I) at either of the tested concentrations. Furthermore, treatment with PSG6 significantly reduced thrombus formation on collagen-coated surfaces, as evidenced by decreases in both thrombus area and integrated fluorescence intensity compared with the vehicle control (Fig. 2J and K). Together, these findings highlight the dual efficacy and safety profile of PSG6.

### 3.2 PSG6 disrupts mitochondrial homeostasis in platelets

**Fig. 3:**
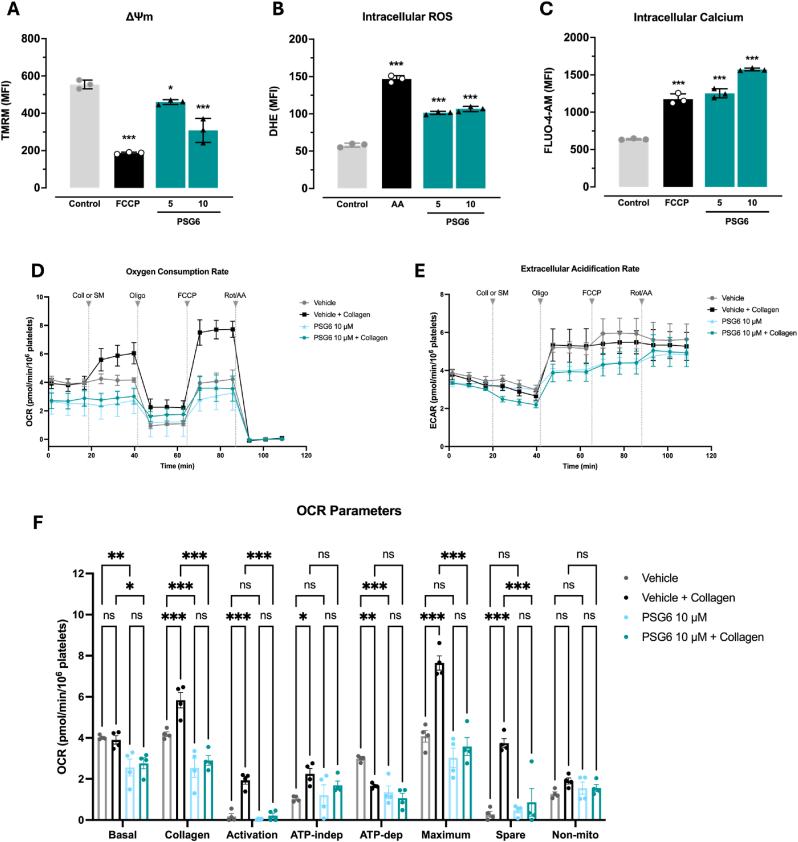
**PSG6 disrupts platelet mitochondrial homeostasis and respiration.***(A) Mitochondrial membrane potential–TMRM/PE. (B) Intracellular ROS–DHE/PE. (C) Intracellular calcium–Fluo4-AM/FITC*. *(D) Oxygen consumption rate (OCR). (E) Extracellular Acidification Rate (ECAR). (F) OCR parameters. Data is shown as mean ± SEM (A-C: n = 3; D-F, n = 4). One-way ANOVA (Bonferroni test) was performed as a statistical analysis for the experiments on A-C. ∗p < 0.05; ∗∗∗p < 0.001 vs vehicle (DMSO 0.2%), and Two-way ANOVA multiple comparisons (Bonferroni test) were used on F. ∗p < 0.05; ∗∗p < 0.01; ∗∗∗p < 0.001 vs*. *control. Coll: Collagen; SM: Seahorse medium; Oligo: Oligomycin; FCCP: Carbonyl cyanide-p-trifluoromethoxyphenylhydrazone; Rot + AA: Rotenone + Antimycin A*.

To evaluate the effects of PSG6 on platelet mitochondrial homeostasis, we analysed key functional parameters, including mitochondrial membrane potential, intracellular calcium levels, and reactive oxygen species (ROS) generation. Incubation with 5 and 10 μM PSG6 induced a dose-dependent decrease in mitochondrial membrane potential (Fig. 3A and Supplementary Fig. 9). In contrast, overall ROS production and intracellular Ca^2+^ levels tended to be elevated compared with the vehicle control (Fig. 3B and C, and Supplementary Figs. 10 and 11, respectively). Mitochondrial respiration was further assessed using the Seahorse XFe24 Extracellular Flux Analyzer. As shown in Fig. 3D–F, the effect of PSG6 on oxygen consumption rate (OCR) was evaluated under basal (non–stimulated) and activated (collagen–stimulated) conditions. PSG6–in either basal or activated states– exhibited a consistent reduction of OCR across multiples parameters. Notably, treatment with 10 μM of PSG6 significantly decrease basal respiration, indicating a direct effect on mitochondrial function under resting conditions. Following collagen stimulation, PSG6 attenuated the expected increase in OCR, with significant reductions observed in collagen-induced respiration, activation, maximal respiration and spare capacity when compared with vehicle-treated activated platelets. Non-mitochondrial respiration remained largely unchanged across conditions. Surprisingly, ATP-linked and proton leak–associated respiration showed only minor, non-significant changes relative to activated controls (Supplementary Table 1). Together, these data support that PSG6 impairs mitochondrial respiration both at baseline and in response to agonist stimulation, supporting a role for mitochondrial modulation in its antiplatelet effects.

### 3.3 PSG6 selectively alters mitochondrial morphology and function in U2OS cells

**Fig. 4:**
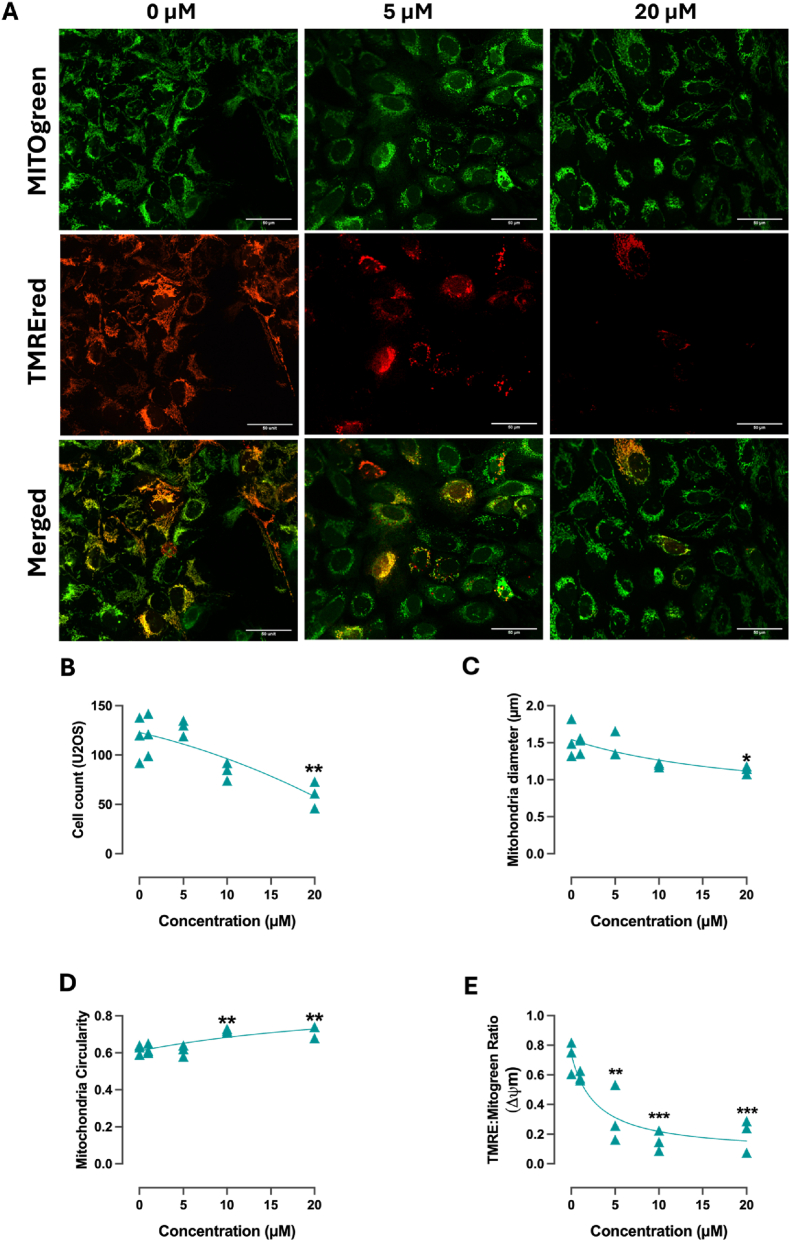
**PSG6 induces mitochondrial morphological changes and depolarization without compromising plasma membrane integrity**. U2OS osteosarcoma cell line stimulated with different concentrations of PSG6 and measured using fluorescence microscopy. (A) From left to right 0 μM, 5 μM and 20 μM; from top to bottom MITOgreen (mitochondrial structure), TRMEred (mitochondrial membrane potential) and Merged (functional mitochondria), (B) A Cell count, (C) Average diameter of the mitochondria, (D) Mitochondrial circularity, (E) Mitochondrial membrane potential normalized over mitochondrial mass. Data is shown as mean ± SEM (n = 3). One-way ANOVA (Bonferroni test) was performed as a statistical analysis. ∗P < 0.05, ∗∗P < 0.01, ∗∗∗P < 0.001.

To further investigate the effects of PSG6 on mitochondrial dynamics, we examined mitochondrial morphology in the U2OS human osteosarcoma cell line (Fig. 4). A clear concentration-dependent decrease in cell number was observed (Fig. 4B). At the mitochondrial level, PSG6 treatment led to a marked reduction in mitochondrial diameter (Fig. 4C) and an increase in circularity (Fig. 4D), both indicative of enhanced mitochondrial fission or fragmentation, a phenotype associated to programmed cell death [76]. These morphological alterations were paralleled by a loss of mitochondrial membrane potential (Fig. 4E), reinforcing the notion that PSG6 primarily targets mitochondrial function.

Importantly, despite these pronounced mitochondrial effects, PSG6 did not compromise plasma membrane integrity, a common drawback of other TPP^+^-linked molecules. This observation suggests that PSG6 exerts a selective action confined to mitochondria, rather than causing generalized cellular damage. Collectively, these findings point to a mitochondria-dependent mechanism underlying PSG6 activity, characterized by controlled mitochondrial fragmentation and depolarization without disruption of the plasma membrane, highlighting its potential as a safer mitochondrial-targeted compound.

### 3.4 PSG6 inhibits mitochondrial complex I activity

Considering the ability of PSG6 to inhibit both platelet aggregation and activation (Fig. 2D–F), along with the marked alterations in mitochondrial parameters, we hypothesised that the loss of mitochondrial membrane potential, together with increased calcium release and elevated reactive oxygen species (ROS) production, as well as the oxygen-consumption data (Fig. 3 and Supplementary Table 1), may result from PSG6 inhibiting the ETC, potentially at the level of complex I or complex III.

**Fig. 5:**
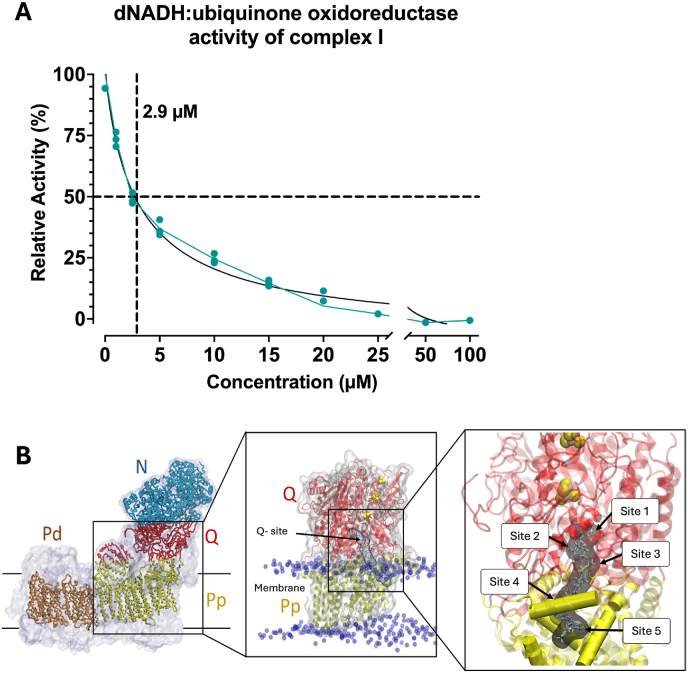
**PSG6 interactions with complex I and its putative binding sites**. (A) Activity assay by DBQ, measures the oxidation of NADH by complex I, dNADH was used as electron donor, DBQ functions as an electron acceptor, DQA inhibits the consumption dependent on complex I (n = 3). (B) Representative of complex I structure and possible binding sites for PSG6.

Given PSG6's hydroquinone structure, a moiety known to interact with the Q-site of complex I, we investigated whether PSG6 could directly inhibit this complex. Using mitochondrial membrane from *Yarrowia lipolytica* (Fig. 5A), we observed that PSG6 completely inhibited dNADH:DBQ oxidoreductase activity at 50 μM (Fig. 5A–Supplementary Table 3), with an IC_50_ of 2.9 μM.

### 3.5 Molecular insights on binding of PSG6 to mitochondrial respiratory complex I

**Fig. 6:**
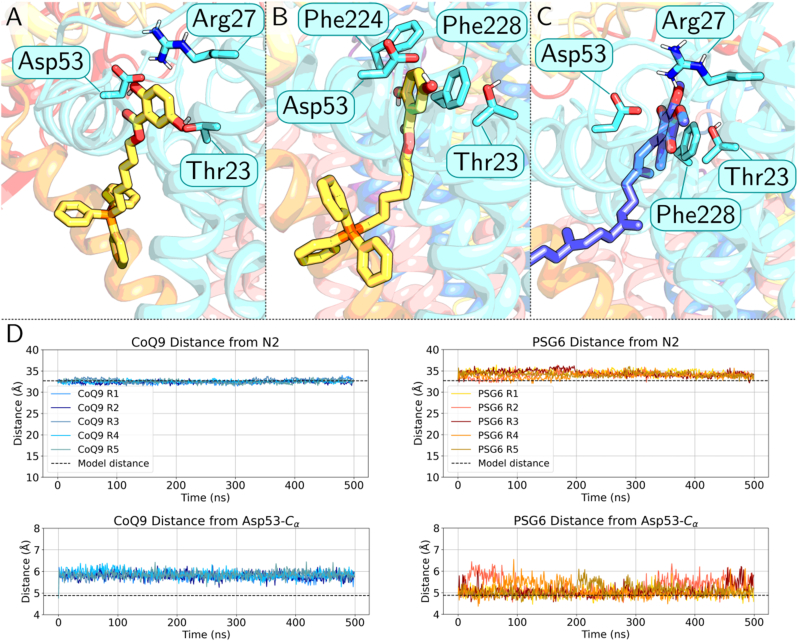
**Molecular dynamics simulations and stability of PSG6 and CoQ9 at shallow binding sites.** (A) Initial docking conformation of PSG6 at sites 4-5 closer to the CoQ tunnel entrance chosen as starting point for MD simulations. PSG6 in yellow sticks representation, in cyan sticks are key ND1 residues within 4 Å of PSG6 phenolic OH groups. Sample PSG6 (B) and CoQ9 (C) conformations from MD simulations. ND1 residues within 4 Å of CoQ9 headgroup carbonyl oxygens are shown in (C). (D) Distance of PSG6 and CoQ9 headgroup from N2 FeS cluster and from Asp53 (Cα atom) during MD simulations. Distance is calculated, for each compound, from five simulations each 500 ns long, with data points sampled every 100 ps. In black dotted line, reference distance taken from initial conformation docked of PSG6.

To obtain molecular insights on how PSG6 may inhibit mitochondrial complex I, we first docked PSG6 to the high-resolution cryo-EM structure of complex I from *Mus musculus* [44]. In this structure, the CoQ tunnel entrance is blocked by an inhibitor derivative that is known for its anticancer properties. We envisaged that PSG6 may bind in a similar location in complex I and therefore docked it to this shallow binding site (see methods). We note that in several high-resolution cryo-EM structures of complex I this region has been observed to trap a ligand molecule [[77], [78], [79]], which supports our docking approach and methodology. Docking calculations showed that PSG6 can bind to the shallow binding sites closer to the entrance of the CoQ tunnel of complex I corresponding to CoQ binding sites 4/5. In this binding pose, the hydroquinonic head group of PSG6 is in the vicinity of polar residues Thr23, Arg27, and Asp53 of the ND1 subunit (Fig. 6A) and 32-34 Å from the N2 FeS cluster (see more below).

**Fig. 7:**
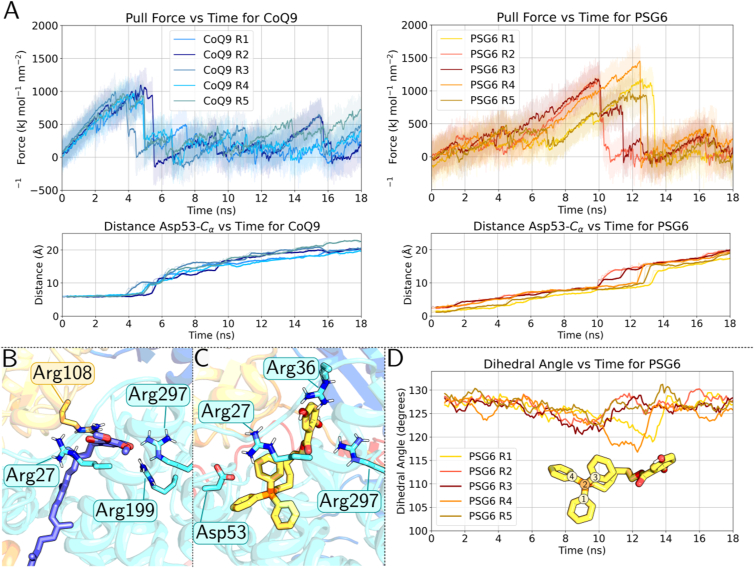
**Steered MD simulations of PSG6 and CoQ9.** (A) Time evolution of the force and distance from Asp53 over the course of pulling simulations of CoQ9 (left) and PSG6 (right). Results for all five replicas are shown. Transparent lines represent raw distance data sampled every 0.1 ps for the force and every 100 ps for the distance, whereas thick solid lines correspond to a rolling average calculated with a window size of 500 points for the force and 5 points for the distance to highlight general trend. Representative CoQ9 (B) and PSG6 (C) conformations corresponding to the peak force in pulling simulations. The arginine residues of the PSST (yellow) and ND1 (cyan) subunits respectively, within 4 Å of CoQ9/PSG6 headgroups, are shown. (D) Improper dihedral angle calculated along all five pulling simulations of PSG6. The angle is calculated considering the atoms 1-2-3-4 highlighted in the figure every 100 ps. Lines correspond to a rolling average calculated with a window size of 8 to highlight general trend.

The binding and dynamics of PSG6 and CoQ9 were explored through atomistic molecular dynamics (MD) simulations (see methods and Supplementary Table 4). Multiple MD simulation replicas (see methods) showed that both the ligands can bind stably to the shallow binding site region of the CoQ tunnel of complex I, albeit in slightly differing positions (Fig. 6B–D). The PSG6 ligand diffuses towards the tunnel entrance and stabilizes in a region where it mostly interacts with residues Asp53 and Thr23, and, via π-stacking with Phe228 or Phe224; an interaction that has been noted in previous studies [80]. On the other hand, CoQ9 ligand holds roughly the same position it is initially modeled in and interacts with polar residues Arg27 and Asp53 of the CoQ tunnel, as shown in Fig. 6. Notably, this CoQ binding arrangement has been observed previously in MD simulations and cryo-EM studies of complex I [45,78,81]. Overall, in ca. 2.5 μs of MD simulations, neither PSG6 nor CoQ9 displayed any significant displacement nor unbinding, with the protein subunits surrounding the ligand as well as the ligand itself displaying low/stable RMSD (Supplementary Fig. 12). These data suggest that PSG6 can bind at the shallow CoQ binding sites of complex I, where several ligands are also known to bind, and this binding mode would in turn prevent the binding of natural substrate CoQ9, thereby limiting enzymatic turnover.

One alternative possibility is that PSG6 may not inhibit complex I by binding at the shallow binding sites of CoQ tunnel, instead it blocks the CoQ cavity by approaching it from alternative routes, such as from the bulk solvent. Indeed, previous MD simulations [45] have identified putative routes through which solvent-bound molecules can reach the CoQ tunnel interior. To this end, chemical biology studies have also suggested that large CoQ-derivatives can reach the redox-active site near the N2 FeS cluster from routes other than the canonical CoQ tunnel [80,82]. Due to the lack of structural information on ligand binding in these alternative routes computational investigation remains a challenge. Instead, we probed with MD simulations if the PSG6 molecule binds the membrane or partitions into the solvent. We performed MD simulations of PSG6 in a protein-free lipid bilayer, corresponding to the inner mitochondrial membrane composition (see Methods). The simulation data show that PSG6 remains bound to the interfacial region of the membrane near the charged lipid headgroups (Supplementary Fig. 13). This is in agreement with the previous free energy simulations on related scaffolds where they have been found to reside in a similar region of the membrane [83,84]. We also note that even though the triphenyl moiety can partition between the hydrophobic core of the membrane and the lipid headgroup region, the hydroquinone group of PSG6 preferentially resides in the region of the membrane where the entry point of CoQ tunnel is (see also [85]). This may provide an energetic advantage for the binding of hydroquinone group to the shallow sites of the CoQ tunnel, whereas binding and access to CoQ tunnel from the solvent exposed routes is likely challenging for a partly lipophilic molecule like PSG6 (approx. consensus logP 5-6 based on [86]).

It is well known that CoQ9 molecule, besides the shallow binding site, also binds to the deep binding site of the CoQ tunnel near the N2 FeS center to undergo redox reaction [87]. However, due to the presence of bulkier triphenyl moiety in the tail of PSG6, it is difficult to envisage how it will bind deeper into the narrow CoQ cavity. Moreover, its diffusion towards the redox active site near N2 from the shallow binding site may also be obstructed. In one previous study involving bulky CoQ-like compound, a strong structural perturbation was observed when the ligand was pulled through the CoQ tunnel with steered MD simulation-like approach [82]. To test if the bulky PSG6 elicits similar behaviour, we initiated non-equilibrium *pulling* (steered) MD simulations and drove CoQ9 and PSG6 inside the CoQ tunnel (Fig. 7, see methods). For both compounds, we observed an increase in force during the pulling simulations. The force build-up observed for CoQ9, corresponds to the situation when the headgroup crosses the segment in the CoQ tunnel rich in positively charged arginine residues (Fig. 7A and B). The data is also shown as the distance between CoQ9 head group and Asp53, which starts to increase from a stable value of ∼6 Å (see also Fig. 6) as CoQ9 head group traverses through the region, which is a well-known CoQ binding site (see above). On the other hand, multiple pulling simulation replicas of PSG6 revealed that the entrance and diffusion of the bulky triphenyl group in the narrow CoQ tunnel is likely obstructed, as implied by the force – time relationship (Fig. 7A). The hydroquinone group of PSG6, which binds closer to the entrance of the CoQ tunnel (and to Asp53, see Fig. 6) reaches the arginine-rich region at around 8-10 ns timeframe (Fig. 7A). At this point, the triphenylphosphonium moiety also enters the narrow CoQ tunnel entrance from the lipid bilayer leading to structural perturbation in the bulky tail group (Fig. 7C). We measured the structural perturbation in the PSG6 tail group with the improper dihedral angle as shown in Fig. 7D, and we observed that the force build-up corresponds to a drop in the dihedral angle of the triphenylphosphonium group. These data suggest that diffusion and binding of PSG6 at the deep binding site is likely energetically unfavorable, instead it may inhibit complex I by targeting the shallow CoQ binding site. However, as noted above, the possibility of PSG6 approaching the deep binding site near N2 from cavities other than the canonical CoQ tunnel cannot be fully eliminated (see Discussion).

## 4 Discussion

Targeting mitochondrial function has emerged as a promising strategy to modulate platelet activity, given the central role of mitochondria in regulating platelet energy metabolism, redox balance, calcium homeostasis, and apoptotic signalling [88,89]. Pharmacological agents designed to selectively interact with platelet mitochondria can influence key processes such as activation, secretion, and aggregation, thereby affecting thrombus formation [23,[90], [91], [92]]. This mitochondria-focused approach offers the potential to fine-tune platelet responses while minimizing systemic effects, positioning mitochondrial modulators as attractive candidates for the development of novel antiplatelet therapies. In this context, our study identifies a mitochondria-targeted complex I inhibitor with selective antiplatelet activity.

We found that the cytotoxic effects of these compounds depend on both their concentration and the length of the linker connecting the TPP^+^ moiety to the active compound. Compounds PSG8 and PSG10 –the derivatives with the longer linkers showed significant cytotoxicity starting at 10 μM. Conversely, aggregation assays revealed that the shorter-linker derivatives, PSG4 and PSG5, lacked antiplatelet activity (Fig. 2). These observations align with our previous findings that linker length is a key determinant of both antiplatelet potency and cytotoxicity: longer linkers tend to increase cytotoxicity, whereas shorter ones fail to confer antiplatelet efficacy [13]. Consistent with this, Kafkova et al. (2023) showed that TPP^+^ cations with a 6-carbon linker selectively and reversibly disrupt protein complexes in the mitochondrial inner membrane [11]. Interestingly, when breast cancer and non-tumourigenic breast cell lines were treated with PSG6, the compound displayed marked selectivity for the cancer cell lines, reducing significantly their viability. This observation aligns with studies showing that metabolically demanding cells –such as many cancer cells and activated platelets– exhibit a hyperpolarized mitochondrial membrane potential [[93], [94], [95]], making them disproportionately susceptible to lipophilic cations like PSG6 [12,96]. This property likely underlies the preferential activity of PSG6 toward pathological cells and the minimal effects observed in healthy cells with lower mitochondrial membrane potential (Supplementary Fig. 1); however, more experiments are needed to understand the exact mechanism by which PSG6 affects, selectively, the viability of breast cancer cells.

As shown previously, PSG6 does not impair clot formation or prolong bleeding time, yet it effectively reduces thrombus formation under flow conditions *in vitro* (Fig. 2). This profile makes PSG6 an attractive candidate for antiplatelet therapy, particularly given that current treatments are often limited by increased bleeding risk and the emergence of drug resistance [[97], [98], [99], [100], [101]].

Accumulating evidence highlights the critical role of mitochondrial function in platelet activation. Because platelets rely predominantly on oxidative phosphorylation, rather than glycolysis, to meet the energetic demands of activation [[102], [103], [104]], and require rapid calcium (Ca^2+^) mobilization for signalling [[105], [106], [107], [108], [109]], the loss of mitochondrial membrane potential observed in PSG6-treated platelets and U2OS cells, could account for the increased intracellular ROS and Ca^2+^ levels, as well as the functional inhibition and morphological changes described above. This is consistent with the fact that mitochondrial ATP production and redox balance are essential for granule secretion, cytoskeletal remodelling, and clot retraction [89,[110], [111], [112]]. The mitochondrial respiration profile of platelets treated with PSG6 shows a significant reduction in collagen-induced OCR, maximum OCR, and spare respiratory capacity, along with a modest, though not statistically significant, decrease in basal OCR. This pattern is consistent with inhibition of one of the electron transport chain (ETC) complexes, likely reflecting a direct effect of PSG6 on mitochondrial function.

Indeed, as demonstrated in our experiments using systems such as *Y. lipolytica* and supported by our docking and classical MD simulations, PSG6 inhibits complex I by binding to its shallow ubiquinone-binding site, thereby suppressing its activity. Accumulating evidence indicates that complex I inhibition impairs mitochondrial respiration and increases oxidative stress, contributing to mitochondrial dysfunction. These alterations can reduce platelet activation and aggregation, consistent with the functional defects observed in PSG6-treated platelets [90,92,[113], [114], [115], [116], [117], [118]]. Consequently, targeting platelet mitochondrial function via complex I inhibition represents a promising strategy for developing novel antiplatelet therapies.

Mitochondrial respiratory complex I, including complexes from yeast mitochondria, are known to display a unique transition where they can convert between two forms: active (A) and deactive (D) [119]. Deactivation of mammalian complex I has been suggested to be a mechanism to prevent excessive ROS production in mitochondria [120]. Based on biochemical studies it has been demonstrated that yeast mitochondrial complex I from *Y. lipolytica* upon purification is predominantly in the D state [78]; a notion also supported by biochemical analysis of other species [51]. The high-resolution cryo-EM structure used to perform MD simulations also displays features that are commensurate with the D state (e.g. π helix in TMH3 of ND6 subunit and arrangement of TMH5-6 of ND1 subunit). In the D (or open-like) states of complex I, the tunnel-bound ligands including CoQ preferentially reside in the shallow regions of the CoQ tunnel (see e.g. Refs. [77,121], which is in line with our docking and simulations of PSG6 (and CoQ9). However, in the absence of structural data on PSG6-bound complex I uncertainty remains, and it is possible that PSG6 approaches the CoQ cavity from alternative routes. For instance, it may partition into the solvent and approach the CoQ tunnel from aqueous phase. Indeed, 3D structures and computer simulations of complex I have highlighted the presence of tunnels that connect the CoQ cavity with the bulk aqueous phase through which ligands could diffuse (see Ref. [45]and references therein). However, due to the absence of ligand densities in cryo-EM structures, it remains unclear if these cavities transport molecules or are just used as proton transfer conduits [87]. Chemical biology studies have also advocated that large CoQ-based derivatives may enter CoQ tunnel from alternative routes and undergo redox reaction [80,82]. Further structural and functional work will be required to prove (or disprove) the proposed binding and inhibition mechanism of PSG6. Our current experimental, and modelling and simulation data suggest that this inhibition can occur by binding of PSG6 to the CoQ tunnel entrance sites.

A limitation of the present study lies in the experimental models and sample size used. Mitochondrial morphology and dynamics were assessed exclusively in the U2OS cell line, which, although widely used for imaging due to its flat morphology and well-defined mitochondrial network [[122], [123], [124]], may not fully recapitulate mitochondrial behaviour in other cell types, including primary cells. Therefore, the generalizability of these observations would benefit from validation in additional cellular models. Furthermore, some experiments displayed variability in the number of biological replicates, and overall sample sizes were relatively small (n = 3–6), which may limit statistical power and the robustness of certain conclusions. While the observed effects were consistent across independent experiments, increasing the number of replicates and extending validation to independent cohorts will be important to strengthen the reproducibility and translational relevance of these findings.

While PSG6 shows promise as a selective antiplatelet agent with minimal effects on bleeding, several questions remain. The precise mechanisms underlying its preferential activity toward metabolically hyperactive cells, including cancer cells and activated platelets, require further elucidation. Additionally, the long-term effects of complex I inhibition on platelet function and systemic metabolism are not yet fully understood. Future studies should explore the therapeutic potential of PSG6 *in vivo*, evaluate possible off-target effects and bioavailability, and determine whether combining mitochondrial-targeted compounds with existing antiplatelet therapies could enhance efficacy while minimizing bleeding risk. Investigating the structure–activity relationship of linker length in TPP^+^ derivatives may also enable optimization of antiplatelet potency while reducing cytotoxicity.

## 5 Conclusion

Our findings demonstrate that PSG6 selectively inhibits platelet activation and aggregation by targeting mitochondrial complex I, without impairing normal haemostasis. The compound's preferential activity toward hyperpolarized mitochondria highlights the potential of mitochondria-targeted strategies for antiplatelet therapy. These results support further development of PSG6 as a novel therapeutic candidate and underscore the critical role of mitochondrial function in regulating platelet-mediated thrombosis and vascular health.

## CRediT authorship contribution statement

**Francisca Tellería:** Investigation. **Matías Monroy-Cárdenas:** Investigation. **Cristina Pecorilla:** Data curation, Formal analysis, Investigation, Writing – review & editing. **Amina Djurabekova:** Formal analysis, Investigation. **Diego Méndez:** Investigation. **Magdalena Sepúlveda:** Investigation. **Felipe Lagos:** Investigation. **Santiago Mansilla:** Investigation. **Laura Castro:** Investigation. **Andrés Trostchansky:** Investigation. **Adriana Covarrubias-Pinto:** Investigation. **Alexis González:** Investigation. **Ivan Dikic:** Investigation. **Iván Palomo:** Investigation. **Volker Zickermann:** Investigation. **Vivek Sharma:** Formal analysis, Funding acquisition, Investigation, Methodology, Supervision, Writing – review & editing. **Lisandra Morales-Malvarez:** Investigation. **Héctor Montecino-Garrido:** Investigation. **Ramiro Araya-Maturana:** Formal analysis, Investigation, Methodology, Writing – original draft, Writing – review & editing. **Eduardo Fuentes:** Formal analysis, Funding acquisition, Investigation, Methodology, Supervision, Writing – original draft, Writing – review & editing.

## Declaration of competing interest

The authors declare that they have no known competing financial interests or personal relationships that could have appeared to influence the work reported in this paper.
