# Supplementary material for: PSG6: A mitochondrially-targeted gentisic acid derivative exerts antiplatelet action via mitochondrial complex I inhibition
Source: Redox Biol. 2026 May 6;94:104200. doi: 10.1016/j.redox.2026.104200 (PMC13202023; doi:10.1016/j.redox.2026.104200)
Supplement: Multimedia component 1 [file mmc1.docx]

**SUPPLEMENTARY INFORMATION**

**PSG6: A MITOCHONDRIALLY-TARGETED GENTISIC ACID DERIVATIVE EXERTS ANTIPLATELET ACTION VIA MITOCHONDRIAL COMPLEX I INHIBITION**

Francisca Tellería^1&^, Matías Monroy-Cárdenas^2,a&^, Cristina Pecorilla^3^, Amina Djurabekova^3,b^, Diego Méndez^1^, Magdalena Sepúlveda^1^, Felipe Lagos^1^, Santiago Mansilla^4^, Laura Castro^5^, Andrés Trostchansky^5^, Adriana Covarrubias-Pinto^6^, Alexis González^6^, Ivan Dikic^6^, Iván Palomo^1^, Volker Zickermann^6^, Vivek Sharma^3,7^, Lisandra Morales-Malvarez^1^, Héctor Montecino-Garrido^1^, Ramiro Araya-Maturana^2*^, Eduardo Fuentes^1*^

^&^These authors contributed equally to this work

^1^Thrombosis and Healthy Aging Research Center, VITALIS Longevity Center, Medical Technology School, Department of Clinical Biochemistry and Immunohematology, Faculty of Health Sciences, Universidad de Talca, Talca, Chile.

^2^Instituto de Química de Recursos Naturales, Universidad de Talca, Talca, Chile. ^a^Current address: Escuela de Química, Facultad de Química y de Farmacia, Pontificia Universidad Católica de Chile, Av. Vicuña Mackenna 4860, Macul, Santiago 7820436, Chile.

^3^Department of Physics, University of Helsinki, Helsinki, Finland. ^b^Current address: Max Planck Institute of Biophysics, Frankfurt am Main, Germany.

^4^Departamento de Métodos Cuantitativos and Centro de Investigaciones Biomédicas (CEINBIO), Facultad de Medicina, Universidad de la República, Montevideo 11800, Uruguay.

^5^Departamento de Bioquímica and Centro de Investigaciones Biomédicas (CEINBIO), Facultad de Medicina, Universidad de la República, Montevideo 11800, Uruguay.

^6^Institute of Biochemistry II, University Hospital, Goethe University, Frankfurt am Main, Germany.

^7^HiLIFE Institute of Biotechnology, University of Helsinki, Helsinki, Finland.

*Correspondence:

Ramiro Araya-Maturana: raraya@utalca.cl

Eduardo Fuentes: edfuentes@utalca.cl

**SUPPLEMENTARY METHODS**

***Synthesis of bromoalkyl-2,5-dihydroxybenzoates (gentisic acid bromoalkyl esters)***. In a 10 mL Monowave 50 reactor process vial, equipped with a magnetic stir bar, 2,5-dihydroxybenzoic acid (500 mg), 1 equivalent of respective alkyl dibromide and 450 µL of triethylamine in acetonitrile (3 mL) were heated for 30 min. at 140 °C, then cooled to room temperature and evaporated under vacuum (Scheme 1). Afterward, bromoalkyl esters BSG4-BSG10 were purified by flash chromatography with ethyl acetate: hexane 1:4.

*4-bromobutyl 2,5-dihydroxybenzoate* (1): Yield (66 %). ^1^H-NMR, δ: 2.04 (m, 4H), 3.48 (t, *J* = 6.3 Hz, 2H), 4.38 (t, *J* = 6.1, 2H), 5.08 (s, OH), 6.89 (d, *J* = 8.9 Hz, 1H), 7.02 (dd, *J1* = 8.8 Hz*, J2* = 3.1 Hz, 1H), 7.28 (d, *J* = 3.1 Hz, 1H), 10.37 (s, OH). ^13^ C-NMR, δ: 27.17, 29.19, 32.91, 64.48, 112.16, 114.63, 118.51, 124.14, 147.75, 155.79, 169.68.

*5-bromopentyl 2,5-dihydroxybenzoate* (1): Yield (62%). ^1^H-NMR, δ: 1.62 (m, 2H), 1.82 (m, 2H), 1.95 (m, 2H), 3.45 (t, *J* = 6.7 Hz, 2H), 4.36 (t, *J* = 6.50 Hz, 2H), 4.81 (s, OH), 6.89 (d, *J* = 8.9 Hz, 1H), 7.02 (dd, *J_1_* = 8.9 Hz*, J_2_* = 3.13 Hz, 1H), 7.29 (d, *J* = 3.13 Hz, 1H), 10.39 (s, OH). ^13^C-NMR, δ: 24.65, 27.73, 32.23, 33.37, 65.14, 112.30, 114.70, 118.55, 124.06, 147.70, 155.92, 169.73. HRMS (ESI) *m/z* calcd. For [C_12_H_15_O_4_Br]CF_3_CO_2_^-^: 415,0010, found: 414,9992.

*6-bromohexyl 2,5-dihydroxybenzoate* (1): White solid with a low melting point. Yield (62%). ^1^HNMR (400 MHz, CDCl_3_) δ: 1.51 (m, 4H), 1.80 (quin, *J* = 7.0 Hz, 2H), 1.90 (*J* = 7.0 Hz, 2H), 3.43 (t, *J* = 6.8 Hz, 2H), 4.35 (t, *J* = 6.6 Hz, 2H), 4.86 (s, OH), 6.89 (d, *J* = 8.9 Hz, 1H), 7.02 (dd, *J_1_* = 9.0 Hz*, J_2_* = 3.1 Hz, 1H), 7.30 (d, *J* = 3.0 Hz, 1H), 10.41 (s, OH). ^13^C-NMR (100 MHz, CDCl_3_) δ: 25.20, 27.75, 28.34, 32.51, 33.71, 65.33, 112.34, 114.68, 118.49, 123.98, 147.68, 155.83, 169.74. HRMS (ESI) *m/z* calcd. For [C_13_H_17_O_4_Br]HCO_2_^-^: 361,0292, found: 361,0296.

*8-bromooctyl 2,5-dihydroxybenzoate.* White solid with a low melting point. Yield (59%). ^1^H-NMR (400 MHz, CDCl_3_) δ: 1.42 (m, 8H), 1.78 (quin, *J* = 7.0 Hz, 2H), 1.87 (quin, *J* = 7.1 Hz, 2H), 3.42 (t, *J* = 6.8 Hz, 2H), 4.34 (t, *J* = 6.6 Hz, 2H), 4.63 (s, OH), 6.89 (d, *J* = 9.0 Hz, 1H), 7.01 (dd, *J_1_* = 8.9 Hz*, J_2_* = 3.2 Hz, 1H), 7.30 (d, *J* = 3.1 Hz, 1H), 10.41 (s, OH). ^13^C-NMR (100 MHz, CDCl_3_) δ: 25.83, 28.01, 28.48, 28.57, 28.98, 32.69, 33.96, 65.50, 112.42, 114.71, 118.50, 123.91, 147.60, 155.93, 169.75. HRMS (ESI) *m/z* calcd. For [C_15_H_21_O_4_Br]CF_3_CO_2_^-^: 457.0473, found: 457.0467.

*10-bromodecyl 2,5-dihydroxybenzoate.* White solid with a low melting point. Yield (65%). ^1^H-NMR (400 MHz, CDCl_3_) δ: 1.40 (m, 12H), 1.77 (quin, *J* = 7.0 Hz, 2H), 1.86 (quin, *J* = 7.1 Hz, 2H), 3.41 (t, *J* = 6.8 Hz, 2H), 4.34 (t, *J* = 6.6 Hz, 2H), 4.72 (s, OH), 6.88 (d, *J* = 8.9 Hz, 1H), 7.01 (dd, *J_1_* = 8.9 Hz*, J_2_* = 3.1 Hz, 1H), 7.30 (d, *J* = 3.1 Hz, 1H), 10.42 (s, OH). ^13^C-NMR (100 MHz, CDCl_3_) δ: 25.94, 28.14, 28.54, 28.71, 29.16, 28.32, 29.36, 32.80, 34.05, 65.61, 112.48, 114.77, 118.49, 123.92, 147.64, 155.92, 169.80. HRMS (ESI) *m/z* calcd. For [C_17_H_25_O_4_Br]CF_3_CO_2_^-^: 485.0786, found: 485.0796.

***Synthesis of phosphonium salts***. To a 10 mL Monowave 50 reactor process vial, equipped with a magnetic stir bar, a mixture of 500 mg of respective 2,5-dihydroxybenzoate and 2,5 eq. of triphenylphosphine in Toluene (3 mL) was heated at 160°C for 30 min. After reaction completion, the mixture was cooled to room temperature and then was evaporated under vacuum. Afterward, phosphonium salts derivatives were purified by flash chromatography with ethyl acetate: methanol 4:1.

*4-((2,5-dihydroxybenzoyl)oxy)butyl)triphenylphosphonium bromide (PSG4)*. *)*. White solid with a low melting point. Yield (9%). ^1^H-NMR δ: 1.67 (m, 2H), 1.98 (m, 2H), 3.50 (m, 2H), 4.24 (t, *J* = 5.9 Hz, 2H), 6.61 (d, *J* = 8.9 Hz, 1H), 6.93 (dd, *J_1_* = 8.94 Hz*, J_2_* = 3.0 Hz, 1H), 7.09 (d, *J* = 3.0 Hz, 1H), 7.64 (m, 15H), 8.68 (s, OH), 9.90 (s, OH). ^13^C-RMN δ: 17.72, 20.08 (d, *J_C-P_* = 50.9 Hz, C_α_), 27.44 (d, *J_C-P_* = 17.5 Hz, C_γ_), 63.31, 110.53, 113.13, 116.53, 116.67 (d, *J_C-P_* = 86.5 Hz, C_1_), 123.14, 129.12 (d, *J_C-P_* = 12.4 Hz, C_3_), 132.22 (d, *J_C-P_* = 10.2 Hz, C_2_), 133.86, 148.40, 153.05, 168.26. HRMS (ESI) *m/z* calcd. For [C_29_H_28_O_4_P]^+^: 471.1725, found: 471.1726.

*5-((2,5-dihydroxybenzoyl)oxy)pentyl)triphenylphosphonium bromide* (PSG5). White solid with a low melting point. Yield (14%). ^1^H-NMR δ: 1.68 (m, 4H), 2.06 (m, 2H), 3.87 (m, 2H), 4.18 (t, *J* = 5.6 Hz, 2H), 6.73 (d, *J* = 8.9 Hz, 1H), 6.98 (dd, *J_1_* = 8.9 Hz*, J_2_* = 3.0 Hz, 1H), 7.72 (m, 15H), 8.05 (d, *J* = 3.0 Hz, 1H), 8.59 (s, OH), 10.05 (s, OH). ^13^C-RMN δ: 22.20 (d, *J_C-P_* = 4.4 Hz, C_β_), 22.75 (d, *J_C-P_* = 50.9, C_α_), 27.14 (d, *J_C-P_* = 17.5 Hz, C_γ_), 27.75, 63.64, 111.67, 115.36, 117.44, 118.15 (d, *J_C-P_* = 86.7 Hz, C_1_), 124.59, 130.43 (d, *J_C-P_* = 12.4 Hz, C_3_), 133.58 (d, *J_C-P_* = 10.2 Hz, C_2_), 134.98 (d, *J_C-P_* = 2.9, C_4_), 150.27, 154.35, 170.11. HRMS (ESI) *m/z* calcd. For [C_30_H_30_O_4_P]^+^: 485.1882, found: 485.1889.

*6-((2,5-dihydroxybenzoyl)oxy)hexyl)triphenylphosphonium bromide* (PSG6). White solid with a low melting point. Yield (32%). ^1^H-NMR δ: 1.64 (m, 8H), 3.68 (m, 2H), 4.23 (t, *J* = 5.5 Hz, 2H), 6.70 (d, *J* = 8.9 Hz, 1H), 7.05 (dd, *J_1_* = 8.9 Hz*, J_2_* = 3.00 Hz, 1H), 7.71 (m, 15H), 7.90 (s, 1H), 8.74 (s, OH), 10.25 (s, OH). ^13^C-RMN δ: 22.57 (d, *J* = 4.4 Hz, C_β_), 22.58 (d, *J_C-P_* = 50.9, C_α_), 25.91, 27.91, 29.87 (d, *J_C-P_* = 16 Hz, C_γ_), 64.95, 111.87, 115.18, 117.58, 118.20 (d, *J_C-P_* = 85.7 Hz, C_1_), 124.72, 130.51 (d, *J_C-P_* = 12.4 Hz, C_3_), 133.59 (d, *J_C-P_* = 10.2 Hz, C_2_), 135.05 (d, *J_C-P_* = 3.6, C_4_), 150.16, 154.54, 170.29. HRMS (ESI) *m/z* calcd. For [C_31_H_32_O_4_P]^+^: 499.2038, found: 499.2047.

*8-((2,5-dihydroxybenzoyl)oxy)octyl)triphenylphosphonium bromide* (PSG8))*.* White solid with a low melting point. Yield (22%). ^1^H-NMR δ: 1.33 (m, 4H), 1.60 (m, 8H), 3.62 (m, 2H), 4.27 (t, *J* = 5.7 Hz, 2H), 6.71 (d, *J* = 8.9 Hz, 1H), 7.11 (dd, *J_1_* = 8.9 Hz*, J_2_* = 3.00 Hz, 1H), 7.72 (m, 16H), 8.77 (s, OH), 10.29 (s, OH). ^13^C-RMN δ: 22.52 (d, *J_C-P_* = 4.4 Hz, C_β_), 22.55 (d, *J_C-P_* = 50.1, C_α_), 25.52, 28.09, 28.26, 28.32, 29.78 (d, *J_C-P_* = 16 Hz, C_γ_), 64.92, 111.99, 115.15, 117.54, 117.54, 118.25 (d, *J_C-P_* = 86.5 Hz, C_1_), 124.90, 130.46 (d, *J_C-P_* = 12.4 Hz, C_3_), 133.55 (d, *J_C-P_* = 10.2 Hz, C_2_), 134.97 (d, *J_C-P_* = 2.9, C_4_), 149.9, 154.60, 170.42. HRMS (ESI) *m/z* calcd. For [C_33_H_36_O_4_P]^+^: 527.2351, found: 527.2349.

*10-((2,5-dihydroxybenzoyl)oxy)decyl)triphenylphosphonium bromide* (PSG10)*.* White solid with a low melting point. Yield (14%). ^1^H-NMR δ: 1.22 (m, 8H), 1.39 (m, 2H), 1.56 (m, 4H), 1.68 (m, 2H), 3.48 (m, 2H), 4.24 (t, *J* = 6.1 Hz, 2H), 6.63 (d, *J* = 8.9 Hz, 1H), 7.32 (dd, *J_1_* = 8.9 Hz*, J_2_* = 3.0 Hz, 1H), 7.39 (d, *J_2_* = 3.0 Hz, 1H), 7.71 (m, 15H), 8.46 (s, OH), 10.25 (s, OH). ^13^C-RMN δ: 22.25-22.85 (m, 2C, C_α_ and C_β_), 25.67, 27.94, 28.44, 28.58, 28.64, 28.72, 30.25 (d, *J_C-P_* = 15.3 Hz, C_γ_), 65.17, 112.02, 115.05, 117.31, 118.05 (d, *J_C-P_* = 85.7 Hz, C_1_), 124.84, 130.44 (d, *J_C-P_* = 13.1 Hz, C_3_), 133.44 (d, *J_C-P_* = 9.5 Hz, C_2_), 135.01, 149.75, 154.47, 170.23. HRMS (ESI) *m/z* calcd. For [C_33_H_36_O_4_P]^+^: 555.2664, found: 555.2669.

1. Wei R, Zhou L, He Y, Wang X, Keller P. Effect of molecular parameters on thermomechanical behavior of side-on nematic liquid crystal elastomers. Polymer (Guildf). 2013 Sep 6;54(20):5321–9. doi:10.1016/j.polymer.2013.07.057

**SUPPLEMENTARY FIGURES AND TABLES**


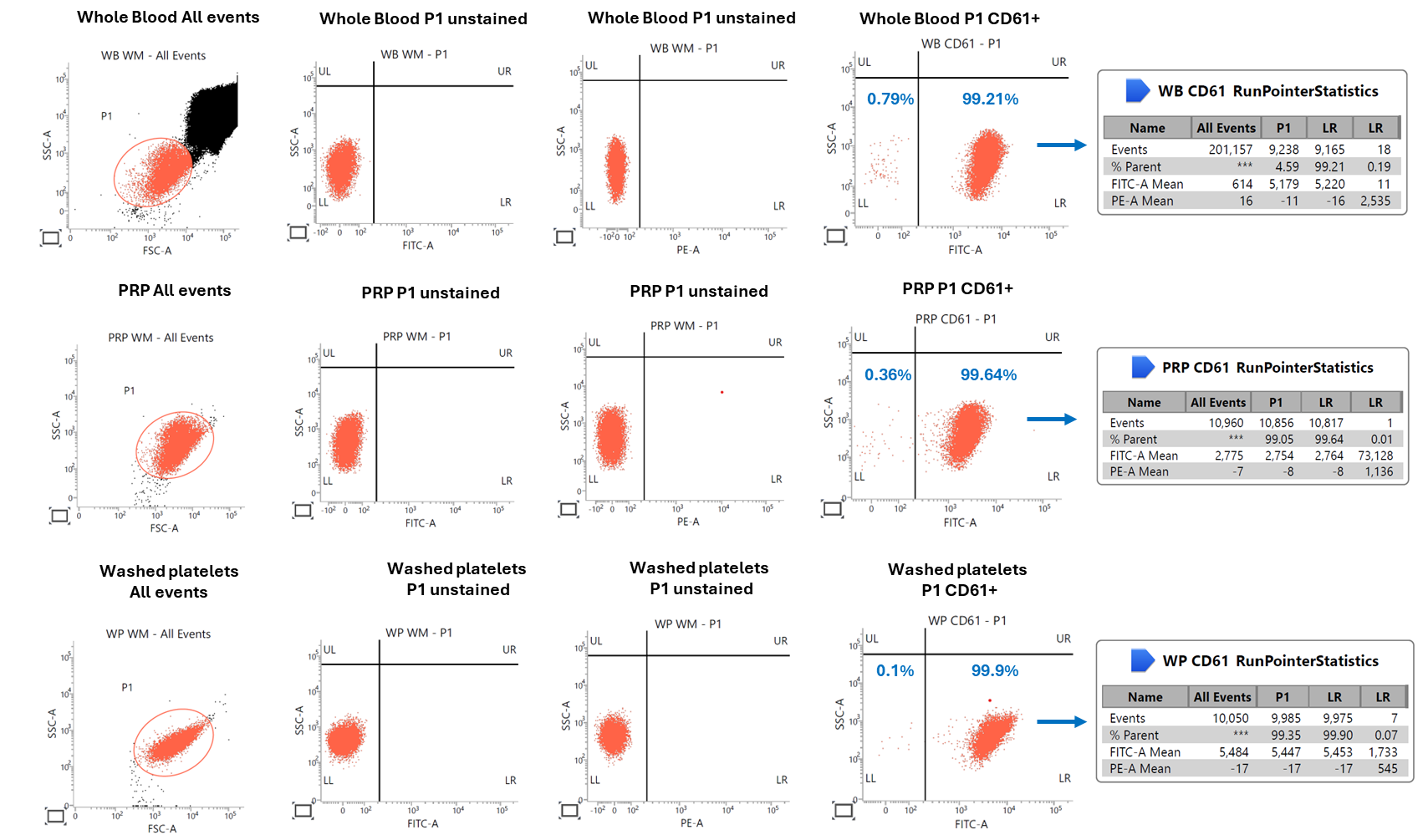


**Supplementary Figure 1. Gating strategies to identify platelet population.** The platelet population was identified using a forward scatter (FSC) versus a side scatter (SSC) dot plot. After selecting the P1 population, platelet purity (>99%) was confirmed using anti-CD61-FITC antibody. Analyses for each technique (probes and antibodies) were conducted by quantifying the mean fluorescence intensity (MFI) or the percentage of positivity for each marker within the P1 CD61+ population. WB: whole blood. PRP: Platelet rich plasma. WP: Washed platelets

***
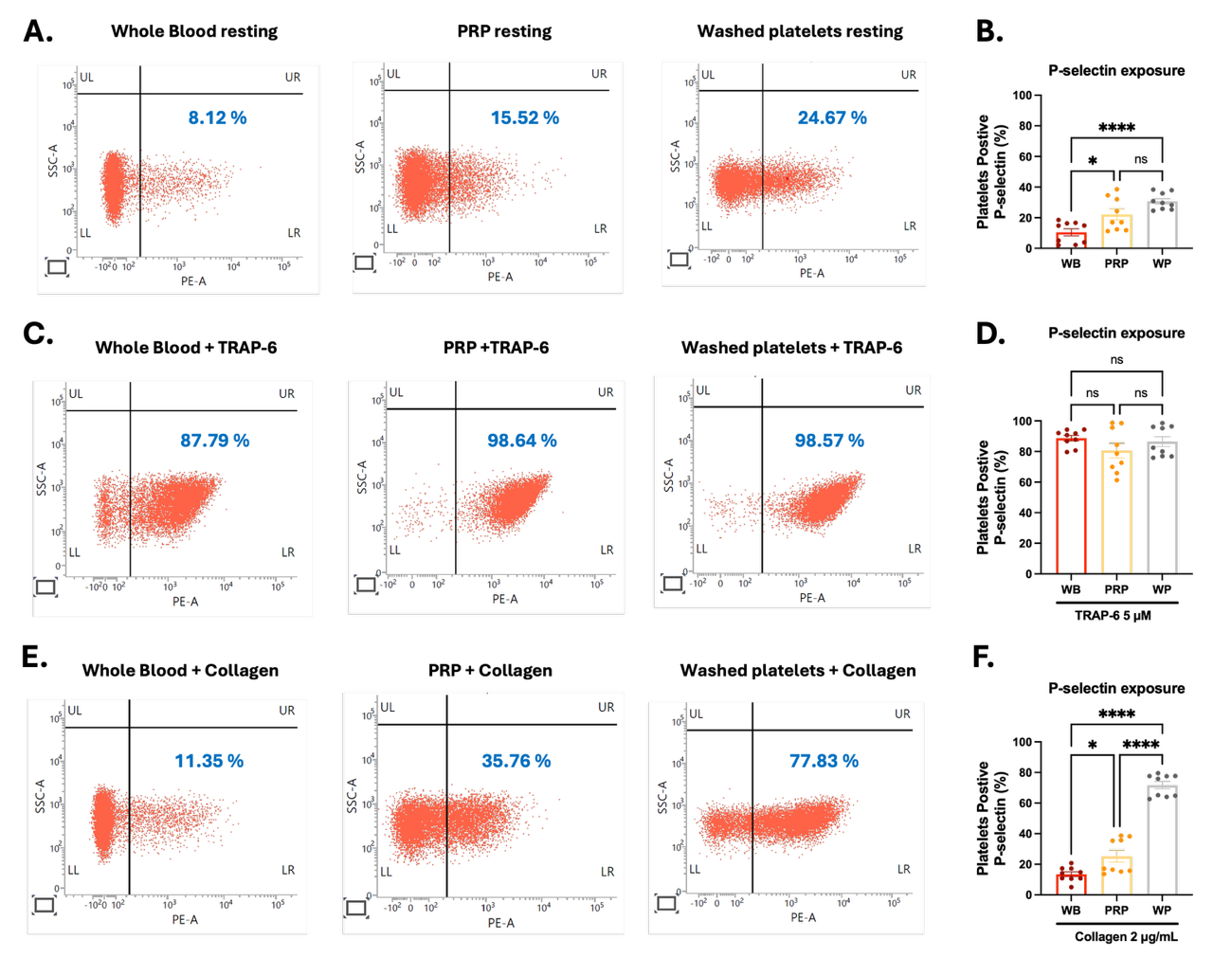
***

**Supplementary Figure 2. Platelet activation controls.** P-selectin levels were quantified in platelets under the following conditions: A-B) Resting; C-D) TRAP-6-activated; E-F) collagen-activated. Results are shown for whole blood (WB, freshly drawn), platelet-rich plasma (PRP), and washed platelets (WP). The bars correspond to the mean ± SEM (n= 9). The statistical analysis was performed using the One-way ANOVA (Bonferroni test). *p < 0.05; ****p < 0.0001.

**Supplementary Table 1.** Oxygen Consumption Rate Parameters.

| Parameters | Vehicle | Vehicle + Collagen | PSG6 10 µM | PSG6 10 µM + Collagen |
| --- | --- | --- | --- | --- |
| **Basal** (OCR/10^6^ platelets) | 4,02 ± 0,05 | 3,91 ± 0,20 | 2,56 ± 0,41 | 2,76 ± 0,27 |
| **Collagen** (OCR/10^6^ platelets) | 4,19 ± 0,11 | 5,84 ± 0,38 | 2,54 ± 0,46 | 2,90 ± 0,24 |
| **Activation** (OCR Collagen – OCR Basal) | 0,18 ± 0,13 | 1,93 ± 0,19 | 0,03 ± 0,02 | 0,21 ± 0,12 |
| **ATP-indep** (OCR/10^6^ platelets) | 1,04 ± 0,07 | 2,25 ± 0,26 | 1,21 ± 0,51 | 1,70 ± 0,20 |
| **ATP-dep** (OCR_Basal_ – OCR_ATP indep_) | 2,99 ± 0,07 | 1,66 ± 0,06 | 1,35 ± 0,31 | 1,06 ± 0,24 |
| **Maximum** (OCR/10^6^ platelets) | 4,08 ± 0,14 | 7,65 ± 0.35 | 3,03 ± 0,47 | 3,58 ± 0,44 |
| **Spare** (OCR_Maximum_ – OCR_Basal_) | 0,28 ± 0,14 | 3,74 ± 0,22 | 0,47 ± 0,13 | 0,86 ± 0,67 |
| **Non-mito** (OCR/10^6^ platelets) | 1,27 ± 0,10 | 1,90 ± 0,15 | 1,55 ± 0,30 | 1,56 ± 0,17 |
| **Coupling efficiency** ((OCR_Basal_ – OCR_ATP-indep_)/OCR_Basal_) | 0,74 ± 0,02 | 0,43 ± 0,04 | 0,56 ± 0,14 | 0,38 ± 0,07 |

**Supplementary Table 2.** Protein chains extracted from *Yarrowia lipolytica* complex I (PDB ID: 7O71) to build the truncated model system for MD simulations. Corresponding subunits in bovine and human complex are mentioned. The amino acid residues modeled with non-standard charge state (doubly protonated histidine, neutral asparatic acid and glutamic acid, and neutral lysine) are described.

| Chain  *Yarrowia lipolytica* | Chain  *Bos taurus* | Chain  Human | Non-standard  charge state |
| --- | --- | --- | --- |
| L | ND4L | NULM | D49  E30  E66 |
| 6 | ND6 | NU6M | K182 |
| 2 | ND2 | NU2M | D39  D69  K282  K383 |
| 5 | ND5 | NU5M | - |
| 3 | ND3 | NU3M | D67  E39  E69 |
| 1 | ND1 | NU1M | E147  E196  E206  E210  E231  K285 |
| C | 49 kDa | NDUFS2 | D196  D331  D363  D465  E151  E376  E379  E463 |
| G | 30 kDa | NDUFS3 | E179  K93 |
| K | PSST | NDUFS7 | - |
| I | TYKY | NDUFS8 | E186 |
| E | 39 kDa | NDUFA9 | H76  D210  E231 |
| F | B13 | NDUFA5 | H108  E92  E109  E110 |
| Z | B14.5a | NDUFA7 | - |
| h | B17.2 | NDUFA12 | H78  D39 |
| P | B14 | NDUFA6 | - |
| U | PGIV | NDUFA8 | - |
| J | B14.7 | NDUFA11 | - |
| W | B16.6 | NDUFA13 | H114  D7  E77 |
| 9 | 15 kDa | NDUFS5 | E53 |
| X | - | - | - |
| g | B9 | NDUFA3 | - |
| D | MWFE | NDUFA1 | H29 |
| b | B14.5b | NDUFC2 | - |
| n | SGDH | NDUFB5 | - |


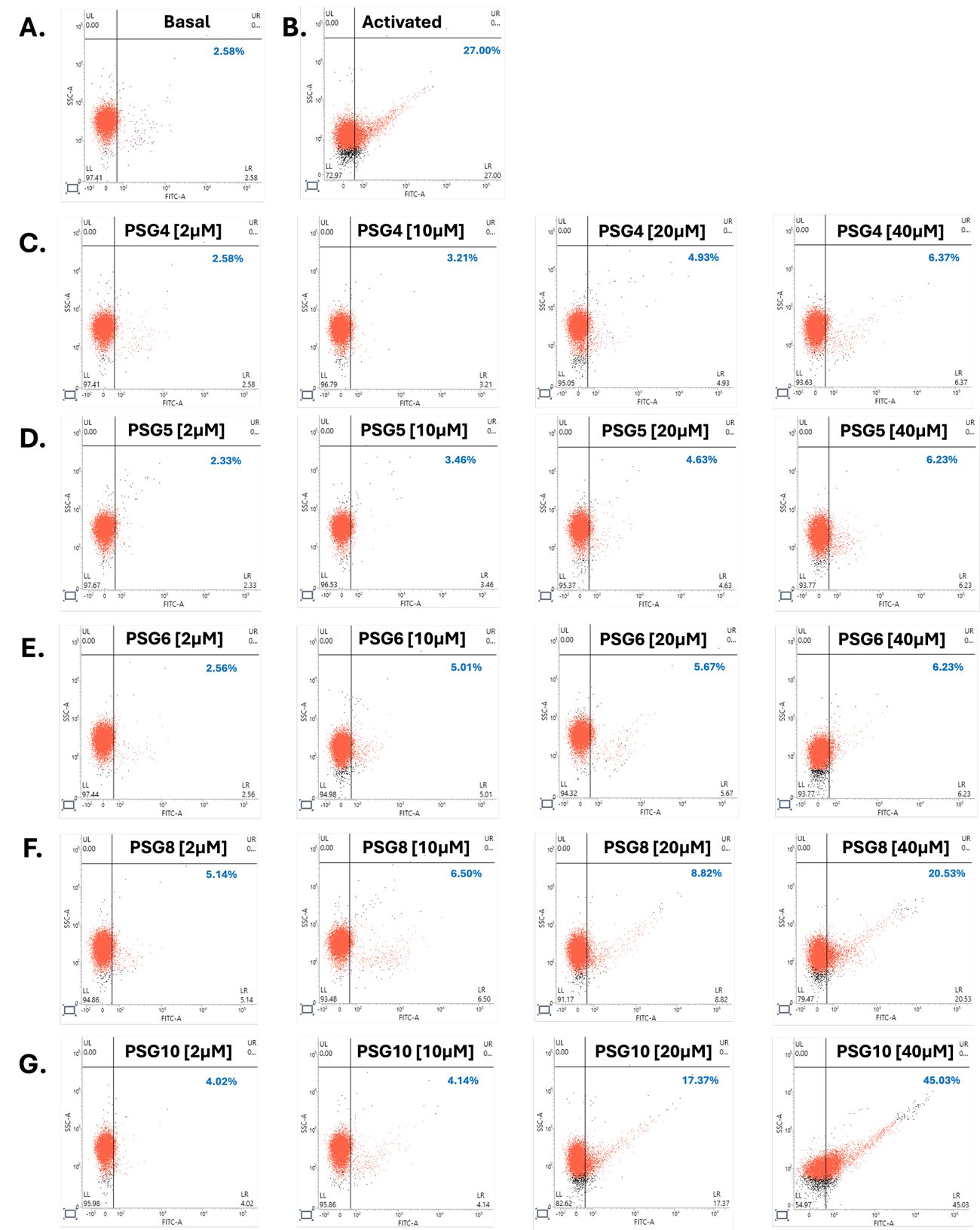


**Supplementary Figure 3. Representative dot plot of platelet apoptosis measured by Annexin-V.** (A) Basal. (B) Triton 0.1%. (C) PSG4. (D) PDG5. (E) PSG6. (F) PSG8. (G) PSG10. (C-G) Platelet were pre-incubated with different concentration of compound (2–40 µM). Percentage of Annexin-V–positive platelets are indicated in blue.


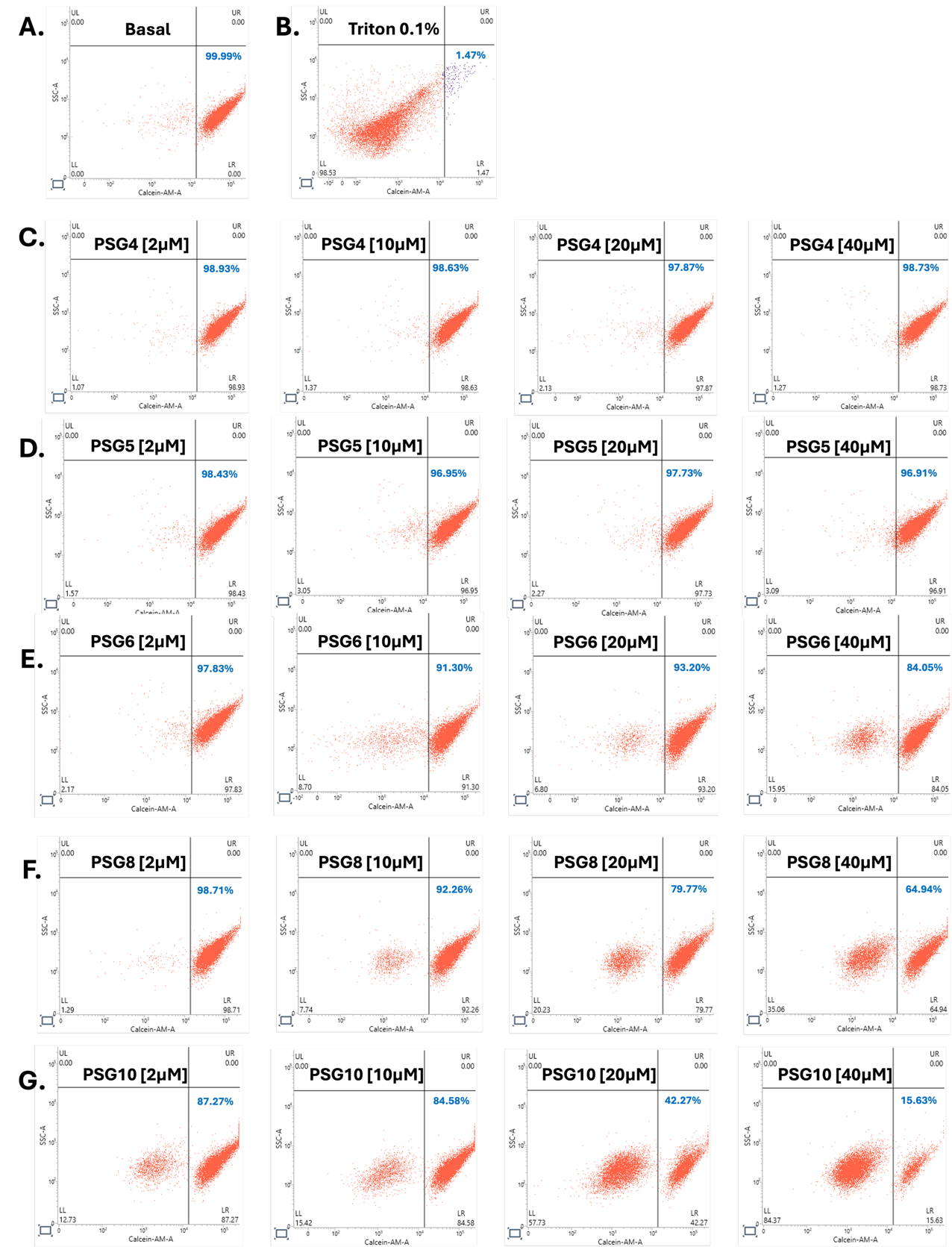


**Supplementary Figure 4. Representative dot plot of platelet viability measured by Calcein-AM.** (A) Basal. (B) Triton 0.1%. (C) PSG4. (D) PDG5. (E) PSG6. (F) PSG8. (G) PSG10. (C-G) Platelet were pre-incubated with different concentration of compound (2–40 µM). Percentage of Calcein-AM–positive platelets are indicated in blue.

**Supplementary Figure 5. Effect of PSG6 on cell viability of tumorigenic and non-tumorigenic breast cell lines**. (A) Cell viability –MTT– when incubated with PSG6 at different concentrations. (B) Cell viability –MTT– of cells whe incubated with PSG6 at 20 µM. RED: MDA-MB-231 –breast cancer cell line–. GREEN: MCF7 –breast cancer cell line–. BLUE: MCF10F –non-tumorigenic cell line–. Data shown as media ± SEM (n=3). One-way ANOVA with Bonferroni’s post hoc test was used for statistical analysis. ***p < 0.001.


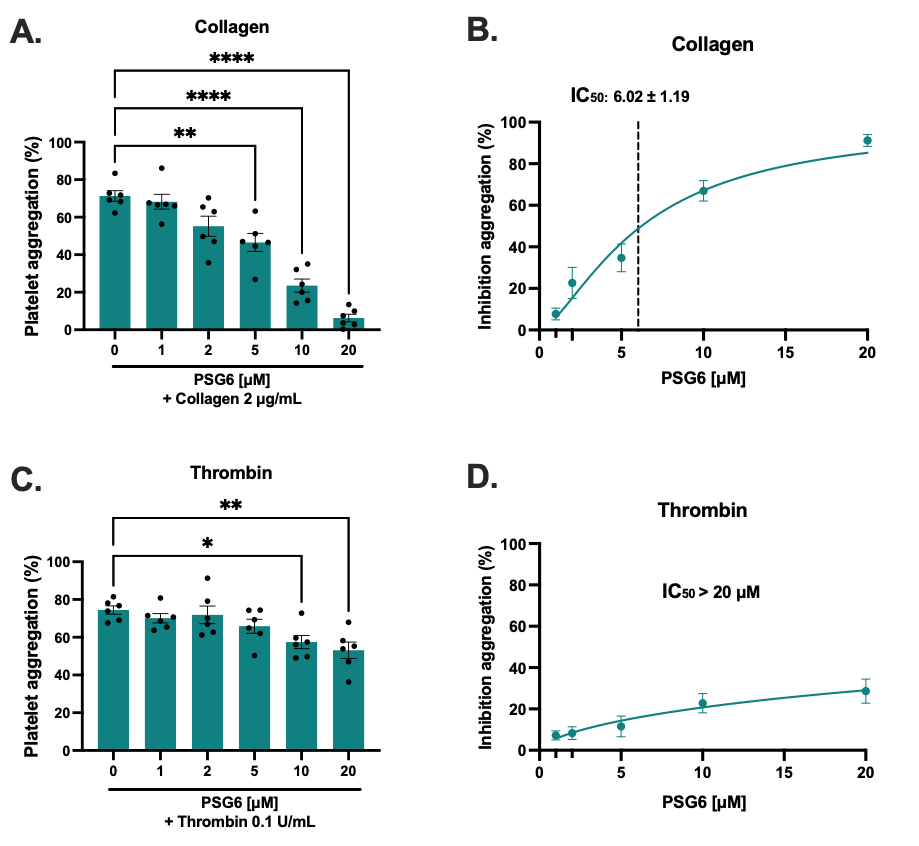


**Supplementary Figure 6. Platelet aggregation with collagen and thrombin.** A) Platelet aggregation collagen. B) IC_50_ collagen. C) Platelet aggregation thrombin. D) IC_50_ thrombin. The bars correspond to the mean ± SEM (n= 6). The statistical analysis was performed using the One-way ANOVA (Bonferroni test). *p < 0.05; **p < 0.01; ****p < 0.0001 vs activated (0). Control: DMSO 0.4% (vehicle). Aggregation was induced using collagen 2 µg/mL or thrombin 0.1 U/mL. IC_50_: Half-maximal Inhibitory Concentration.


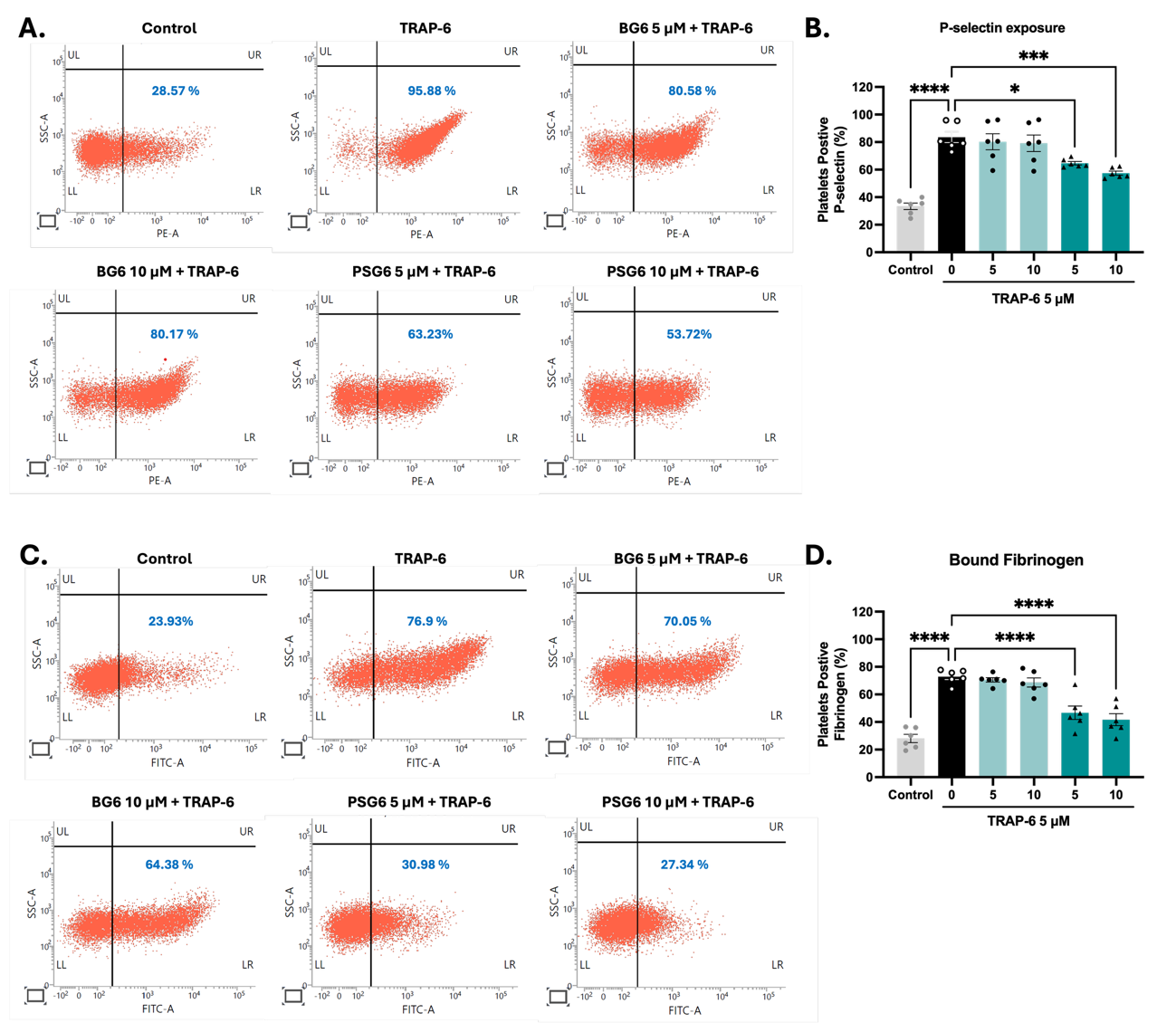


**Supplementary Figure 7. PSG6 inhibits granule secretion and GP IIb/IIIa activation in TRAP-6-stimulated platelets.** A-B) P-selectin levels. C-D) Bound fibrinogen. Results are shown as percentage positivity for each marker in the CD61+ (P1) platelet population. The bars correspond to the mean ± SEM (n= 6). The statistical analysis was performed using the One-way ANOVA (Bonferroni test). *p < 0.05; ***p < 0.001; ****p < 0.0001 vs control or activated (0).


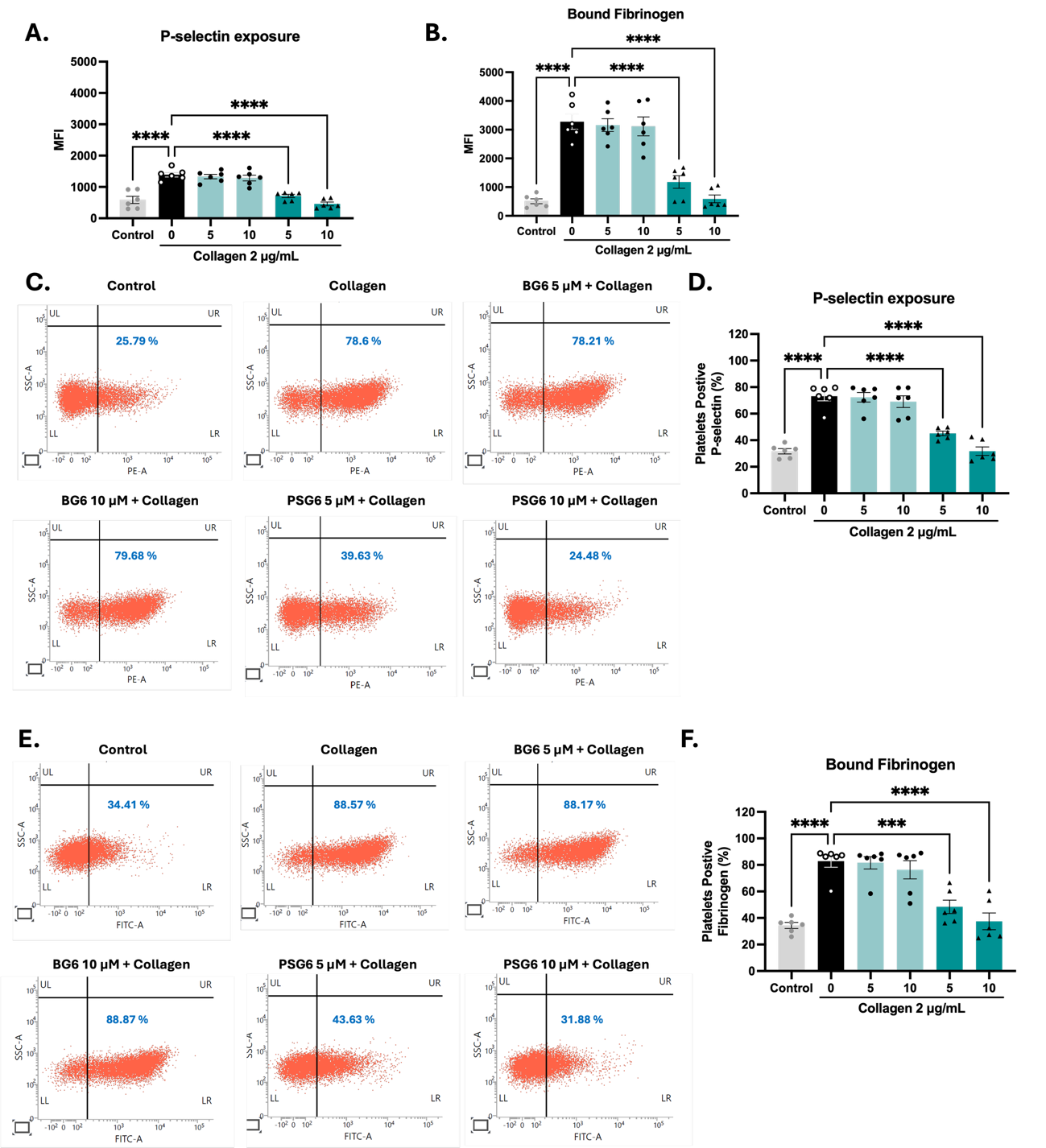


**Supplementary Figure 8. PSG6 inhibits granule secretion and GP IIb/IIIa activation in collagen-stimulated platelets**. A) P-selectin levels (MFI). B) Bound fibrinogen (MFI). C-D) P-selectin (percentage positivity). E-F) Bound fibrinogen (percentage positivity). The percentage of positivity was determined within the CD61+ (P1) platelet population. The bars correspond to the mean ± SEM (n= 6). The statistical analysis was performed using the One-way ANOVA (Bonferroni test). ***p < 0.001; ****p < 0.0001 vs control or activated (0).


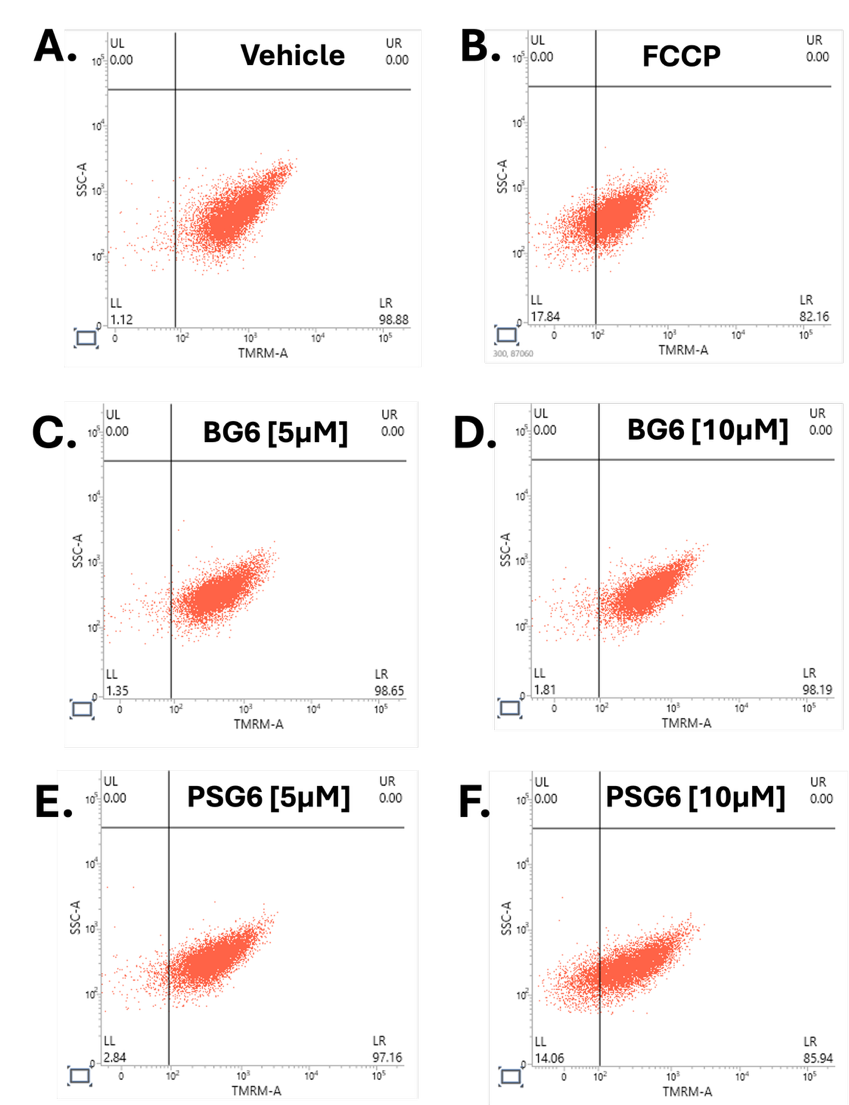


**Supplementary Figure 9. Representative dot plot of platelet mitochondrial membrane potential measured by TMRM/PE probe.** (A) Basal. (B) FCCP 1 µM (C) BSG6 at 5 µM. (D) BSG6 at 10 µM. (E) PSG6 at 5 µM. (F) PSG6 at 10 µM.

**
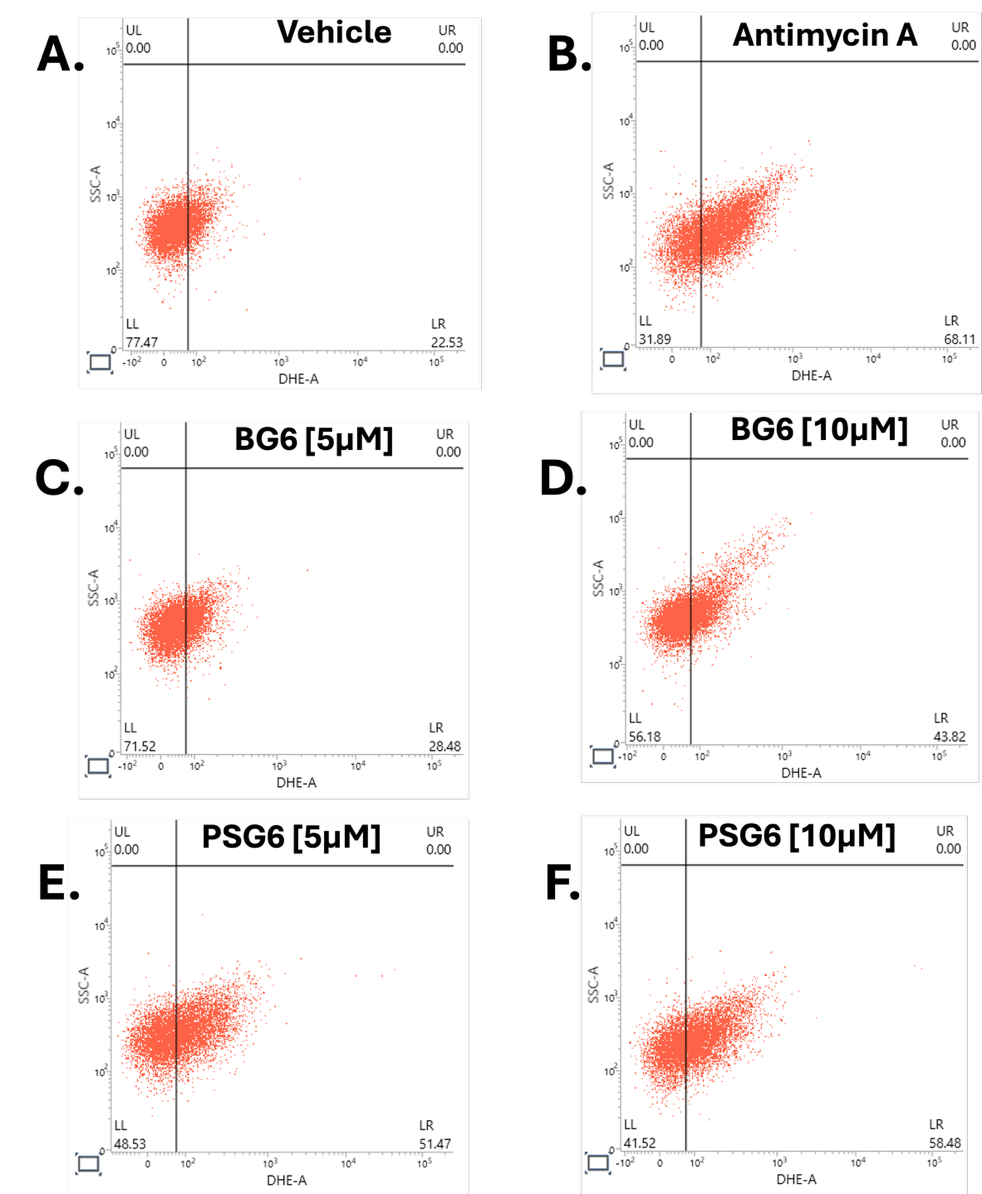
**

**Supplementary Figure 10. Representative dot plot of intracellular ROS production measured by DHE/PE probe.** (A) Basal. (B) Antimycin A 20 µM (C) BSG6 at 5 µM. (D) BSG6 at 10 µM. (E) PSG6 at 5 µM. (F) PSG6 at 10 µM.

***
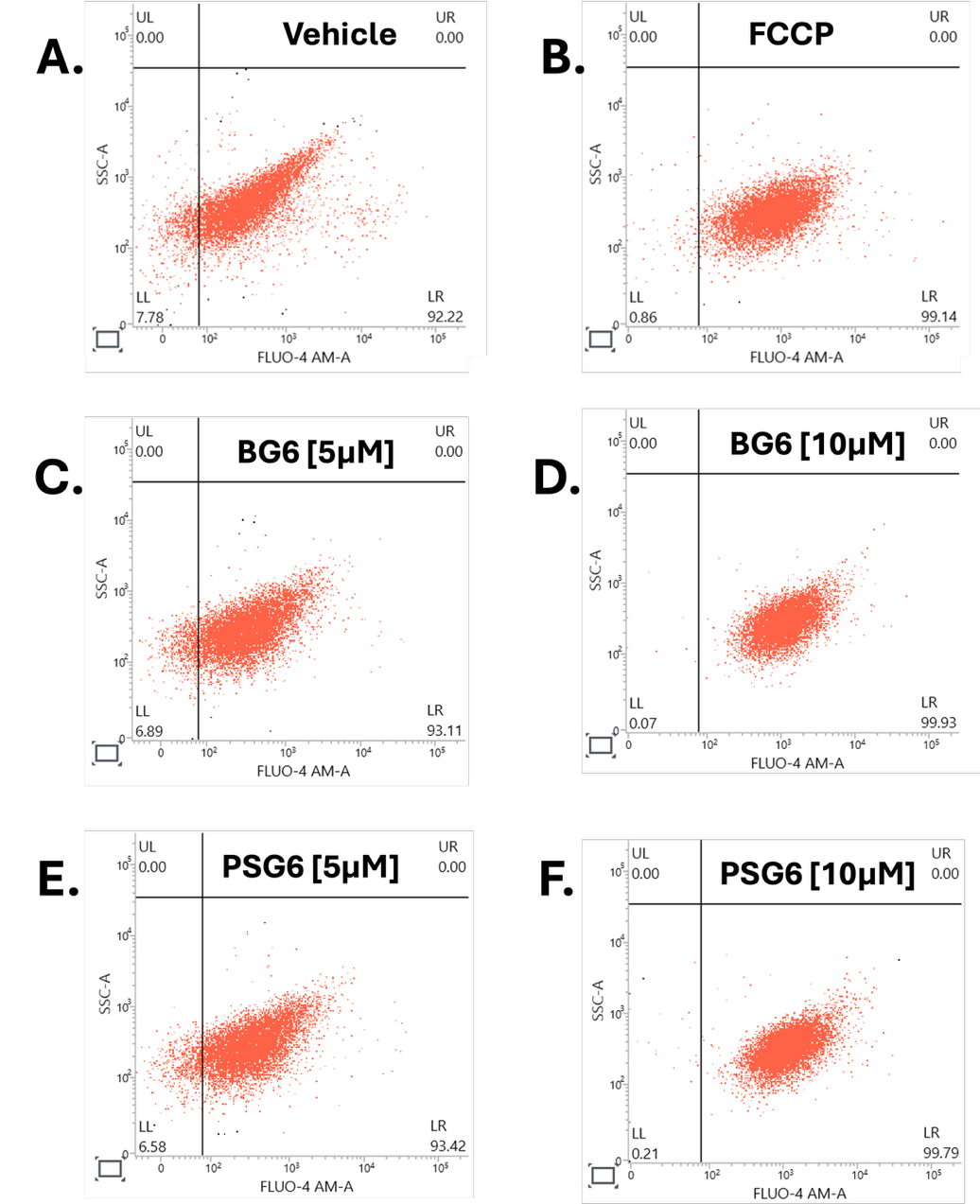
***

**Supplementary Figure 11. Representative dot plot of platelet intracellular calcium levels measured by Fluo-4-AM/FITC probe**. (A) Basal. (B) FCCP 1 µM (C) BSG6 at 5 µM. (D) BSG6 at 10 µM. (E) PSG6 at 5 µM. (F) PSG6 at 10 µM.

**Supplementary Table 3.** dNADH:ubiquinone oxidoreductase activity.

| Condition | Protein  µg/mL | HAR Activity (Mean)  µmol min-1 mg-1 | DBQ - DQA Activity (Mean) | HAR/Protein (U/mg) | DBQ/Protein (U/mg) | DBQ/HAR (Mean) | % Activity |
| --- | --- | --- | --- | --- | --- | --- | --- |
| WT + 0 µM | 52,7 | 44,7 | 34 ,0 | 0,85 | 0,65 | 0,76 | 100 % |
| WT + 1 µM | 52,7 | 44,7 | 25,0 | 0,85 | 0,47 | 0,56 | 73,5 % |
| WT + 2,5 µM | 52,7 | 44,7 | 16,7 | 0,85 | 0,32 | 0,37 | 49,1 % |
| WT + 5 µM | 52,7 | 44,7 | 12,6 | 0,85 | 0,24 | 0,28 | 36,9 % |
| WT + 10 µM | 52,7 | 44,7 | 8,3 | 0,85 | 0,16 | 0,19 | 24,5 % |
| WT + 15 µM | 52,7 | 44,7 | 5,0 | 0,85 | 0,10 | 0,11 | 14,7 % |
| WT + 20 µM | 52,7 | 44,7 | 1,8 | 0,85 | 0,03 | 0,04 | 5,3 % |
| WT + 25 µM | 48,8 | 45,6 | -0,2 | 0,93 | 0,00 | 0,00 | -0,5 % |
| WT + 50 µM | 48,8 | 45,6 | -0,5 | 0,93 | -0,01 | -0,01 | -1,4 % |
| WT + 100 µM | 48,8 | 45,6 | 0,7 | 0,93 | 0,01 | 0,01 | 1,9 % |

*Data for Mean ± SEM, n=3*

**Supplementary Table *4*.** Model systems studied.

| Model System | Ligand | # Simulations | Simulation length (unbiased simulations) | Lipids | Ions | Total number of atoms |
| --- | --- | --- | --- | --- | --- | --- |
| Protein + Membrane | CoQ | 5 unbiased  5 steered | 500 ns | 291 POPC  183 POPE  67 TLCL | 373 Na^+^ 253 Cl^-^ | 543749 |
| Protein + Membrane | PSG6 | 5 unbiased  5 steered | 500 ns | 290 POPC  179 POPE  67 TLCL | 373 Na^+^ 253 Cl^-^ | 543685 |
| Protein + Membrane | PSG6-Br | 5 unbiased | 500 ns | 290 POPC  179 POPE  67 TLCL | 373 Na^+^ 253 Cl^-^ | 543686 |
| Membrane | PSG6 | 3 unbiased | 250 ns | 404 POPC  262 POPE  108 TLCL | 495 Na^+^ 279 Cl^-^ | 558329 |
| Membrane | PSG6-Br | 3 unbiased | 250 ns | 404 POPC  262 POPE  108 TLCL | 495 Na^+^ 279 Cl^-^ | 558330 |


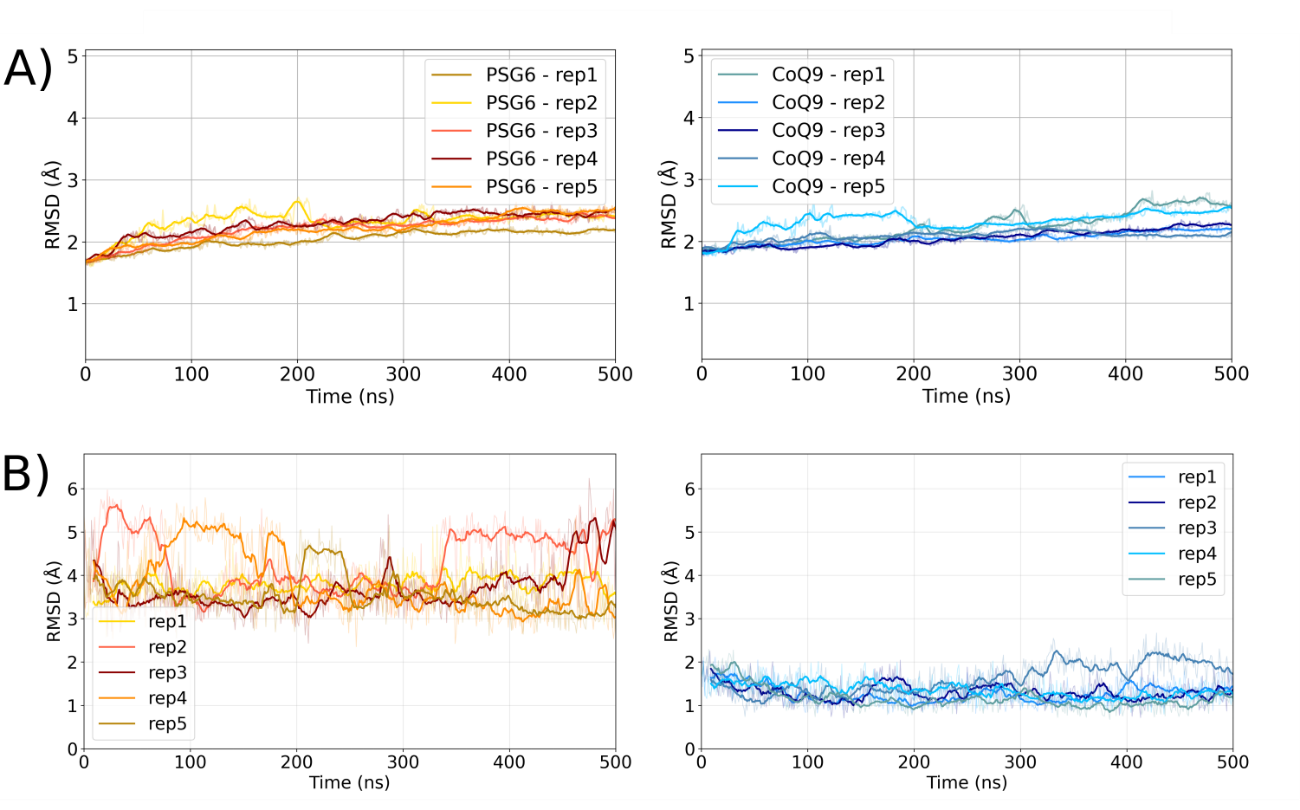


**Supplementary Figure 12.** (A) RMSD of the Yarrowia lipolytica complex I subunits ND1, 49kDa, and PSST subunits in MD simulations (five independent replicas, rep1-5) for PSG6 (left) and CoQ9 (right). (B) RMSD of the PSG6 (left) and CoQ9 (right, only head group) in the same unbiased MD simulations. RMSD of the ligands was calculated after alignment of the protein heavy atoms. For all panels, raw RMSD values are shown as transparent lines, with smoothed rolling averages overlaid as solid lines. For CoQ9, the RMSD of the head group is shown, excluding the long tail, which undergoes large fluctuations due to its mixing with the lipid membrane.

*
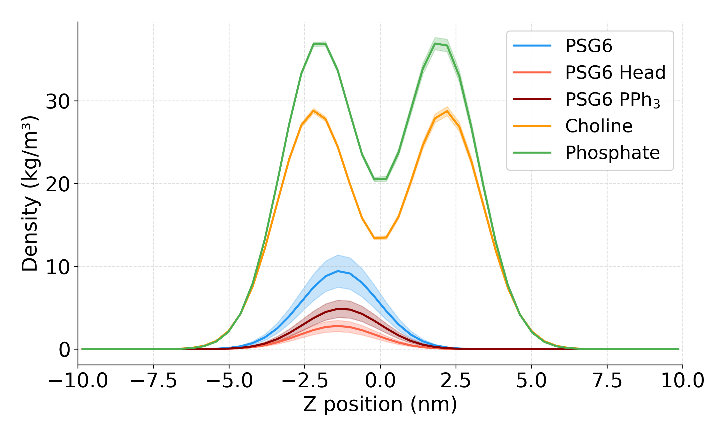
*

**Supplementary Figure 13**. Partial mass density profiles along the membrane normal (Z-axis) for selected components of the PSG6-membrane system, averaged over 3 independent replicas 250 ns long (shaded regions represent standard deviation). Membrane leaflet positions are indicated by the choline (orange) and phosphate (green) group density peaks of POPC. The PSG6 ligand total density (blue), hydroquinone head group density (red), and triphenylphosphorus moiety density (dark red) are scaled by a factor of 10 for visualization purposes due to the low ligand concentration relative to the bulk membrane lipids. Only POPC choline and phosphate groups are shown as membrane reference markers; the full membrane composition includes POPC, POPE, and TLCL.


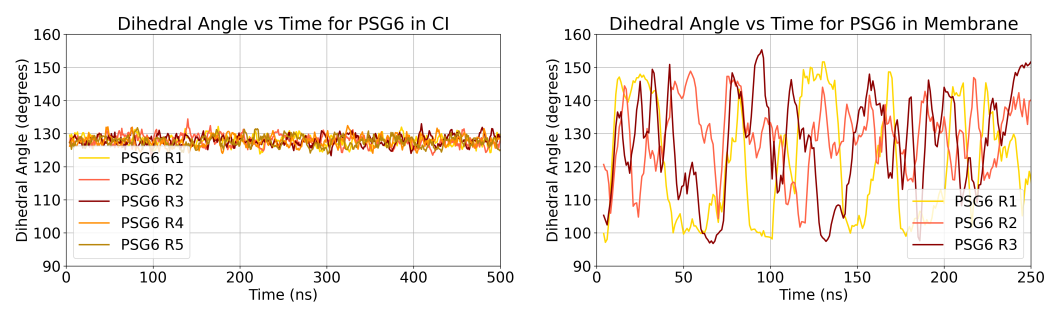


**Supplementary Figure 14**. Improper dihedral angle of PSG6 calculated along five independent unbiased MD simulations in complex I (left) and three independent unbiased membrane simulations (right). In the complex I simulations, the dihedral angle remains stable at ~128°, indicating that the triphenylphosphorus group of PSG6 is constrained due to protein binding. In contrast, the membrane simulations show large fluctuations spanning ~100–160°, reflecting the conformational freedom of PSG6 in a lipid environment. The angle is defined by atoms 1-2-3-4 highlighted in Figure 7, sampled every 100 ps

**SUPPLEMENTARY DATA**

Force field parameters of PSG6 obtained from CHARMM-GUI ligand reader and modeler (see methods).

PSG6_Br.rtf

* Topologies generated by

* CHARMM General Force Field (CGenFF) program version 2.5.1

*

36 1

RESI lig 0.000 ! param penalty= 259.000 ; charge penalty= 325.621

GROUP ! CHARGE CH_PENALTY

ATOM C1 CG2R61 -0.115 ! 0.000

ATOM C2 CG2R61 -0.115 ! 8.270

ATOM C3 CG2R61 -0.245 ! 20.543

ATOM C4 CG2R61 -0.440 ! 188.074

ATOM C5 CG2R61 -0.245 ! 20.543

ATOM C6 CG2R61 -0.115 ! 8.270

ATOM C7 CG2R61 -0.245 ! 20.543

ATOM C8 CG2R61 -0.440 ! 188.074

ATOM C9 CG2R61 -0.245 ! 20.543

ATOM C10 CG2R61 -0.115 ! 8.270

ATOM C11 CG2R61 -0.115 ! 0.000

ATOM C12 CG2R61 -0.115 ! 8.270

ATOM C13 CG2R61 -0.440 ! 188.074

ATOM C14 CG2R61 -0.245 ! 20.543

ATOM C15 CG2R61 -0.115 ! 8.270

ATOM C16 CG2R61 -0.115 ! 0.000

ATOM C17 CG2R61 -0.115 ! 8.270

ATOM C18 CG2R61 -0.245 ! 20.543

ATOM P PG0 1.760 ! 325.621! typer WARNING: non-phosphate non-phosphonate phosphorus not explicitly supported

ATOM C19 CG321 -0.316 ! 41.291

ATOM C20 CG321 -0.177 ! 16.413

ATOM C21 CG321 -0.180 ! 1.045

ATOM C22 CG321 -0.189 ! 0.000

ATOM C23 CG321 -0.169 ! 0.000

ATOM C24 CG321 0.059 ! 0.476

ATOM O1 OG302 -0.295 ! 0.476

ATOM C25 CG2O2 0.448 ! 12.153

ATOM C26 CG2R61 0.222 ! 20.182

ATOM C27 CG2R61 -0.013 ! 20.153

ATOM C28 CG2R61 -0.111 ! 0.000

ATOM C29 CG2R61 -0.112 ! 0.000

ATOM C30 CG2R61 0.108 ! 0.000

ATOM C31 CG2R61 -0.107 ! 0.000

ATOM O2 OG311 -0.545 ! 12.465

ATOM O3 OG311 -0.533 ! 0.000

ATOM H1 HGR61 0.210 ! 13.510

ATOM H2 HGR61 0.115 ! 0.000

ATOM H3 HGR61 0.115 ! 0.000

ATOM H4 HGR61 0.115 ! 0.000

ATOM H5 HGR61 0.210 ! 13.510

ATOM H6 HGR61 0.210 ! 13.510

ATOM H7 HGR61 0.115 ! 0.000

ATOM H8 HGR61 0.115 ! 0.000

ATOM H9 HGR61 0.115 ! 0.000

ATOM H10 HGR61 0.210 ! 13.510

ATOM H11 HGR61 0.210 ! 13.510

ATOM H12 HGR61 0.115 ! 0.000

ATOM H13 HGR61 0.115 ! 0.000

ATOM H14 HGR61 0.115 ! 0.000

ATOM H15 HGR61 0.210 ! 13.510

ATOM H16 HGA2 0.090 ! 4.331

ATOM H17 HGA2 0.090 ! 4.331

ATOM H18 HGA2 0.090 ! 1.000

ATOM H19 HGA2 0.090 ! 1.000

ATOM H20 HGA2 0.090 ! 0.000

ATOM H21 HGA2 0.090 ! 0.000

ATOM H22 HGA2 0.090 ! 0.000

ATOM H23 HGA2 0.090 ! 0.000

ATOM O4 OG2D1 -0.488 ! 0.000

ATOM H24 HGP1 0.420 ! 0.000

ATOM H25 HGP1 0.420 ! 0.000

ATOM H26 HGR61 0.115 ! 0.000

ATOM H27 HGR61 0.115 ! 0.000

ATOM H28 HGR61 0.115 ! 0.000

ATOM H29 HGA2 0.090 ! 0.000

ATOM H30 HGA2 0.090 ! 0.000

ATOM H31 HGA2 0.090 ! 0.000

ATOM H32 HGA2 0.090 ! 0.000

ATOM BR BRGA1 -0.197 ! LP charge added here

!ATOM LP1 LPH 0.050 ! on BR

BOND C1 C2

BOND C2 C3

BOND C3 C4

BOND C4 C5

BOND C5 C6

BOND C6 C1

BOND C7 C8

BOND C8 C9

BOND C9 C10

BOND C10 C11

BOND C11 C12

BOND C12 C7

BOND C13 C14

BOND C14 C15

BOND C15 C16

BOND C16 C17

BOND C17 C18

BOND C18 C13

BOND C4 P

BOND C8 P

BOND C13 P

BOND P C19

BOND C19 C20

BOND C20 C21

BOND C21 C22

BOND C22 C23

BOND C23 C24

BOND C25 C26

BOND C27 C28

BOND C28 C29

BOND C29 C30

BOND C30 C31

BOND C26 C27

BOND C31 C26

BOND C5 H5

BOND C24 O1

BOND O1 C25

BOND O4 C25

BOND C27 O2

BOND C30 O3

BOND H24 O2

BOND H25 O3

BOND C28 H28

BOND C29 H27

BOND C31 H26

BOND H31 C24

BOND H32 C24

BOND H30 C23

BOND H29 C23

BOND H22 C22

BOND H23 C22

BOND H21 C21

BOND H20 C21

BOND H19 C20

BOND H18 C20

BOND H17 C19

BOND H16 C19

BOND H15 C14

BOND H14 C15

BOND H13 C16

BOND H12 C17

BOND H11 C18

BOND H10 C9

BOND H9 C10

BOND H8 C11

BOND H7 C12

BOND H6 C7

BOND C6 H4

BOND C1 H3

BOND C2 H2

BOND C3 H1

!BOND P BR

IMPR C25 C26 O4 O1

!LONEPAIR COLI LP1 BR P DIST 1.8900 SCAL 0.0

END

PSG6_Br.prm

* Parameters generated by analogy by

* CHARMM General Force Field (CGenFF) program version 2.5.1

*

BONDS

CG2R61 PG0 190.00 1.7300 ! lig , from CG2R61 SG3O2, penalty= 195

CG321 PG0 248.40 1.8617 ! lig , from CG331 PG0, penalty= 6

ANGLES

CG2R61 CG2R61 PG0 10.00 122.30 ! lig , from CG2R61 CG2R61 SG3O1, penalty= 20

CG321 CG321 PG0 43.00 105.50 ! lig , from CG321 CG321 SG3O1, penalty= 20

PG0 CG321 HGA2 90.00 110.00 5.40 1.80200 ! lig , from PG1 CG321 HGA2, penalty= 2

CG2R61 PG0 CG2R61 119.54 120.64 ! lig , from NG2S3 PG0 OG2P1, penalty= 114

CG2R61 PG0 CG321 64.36 121.00 ! lig , from CG331 PG0 OG2P1, penalty= 64.9

DIHEDRALS

CG2R61 CG2O2 OG302 CG321 1.2500 1 180.00 ! lig , from CG2R61 CG2O2 OG302 CG331, penalty= 0.9

CG2R61 CG2O2 OG302 CG321 1.5000 2 180.00 ! lig , from CG2R61 CG2O2 OG302 CG331, penalty= 0.9

CG2R61 CG2O2 OG302 CG321 0.0500 6 180.00 ! lig , from CG2R61 CG2O2 OG302 CG331, penalty= 0.9

CG2O2 CG2R61 CG2R61 OG311 2.4642 2 180.00 ! lig , from CG2DC1 CG2R61 CG2R61 OG311, penalty= 28.5

CG2R61 CG2R61 CG2R61 PG0 3.1000 2 180.00 ! lig , from CG2R61 CG2R61 CG2R61 SG3O1, penalty= 20

PG0 CG2R61 CG2R61 HGR61 2.4000 2 180.00 ! lig , from SG3O1 CG2R61 CG2R61 HGR61, penalty= 20

CG2R61 CG2R61 PG0 CG2R61 0.0000 6 0.00 ! lig , from CG2R61 CG2R61 SG3O2 OG2P1, penalty= 259

CG2R61 CG2R61 PG0 CG321 0.2200 2 0.00 ! lig , from CG2R61 CG2R61 SG3O2 NG311, penalty= 234.5

CG321 CG321 CG321 PG0 0.9400 1 180.00 ! lig , from CG331 CG321 CG321 SG3O1, penalty= 20.9

CG321 CG321 CG321 PG0 0.3800 2 0.00 ! lig , from CG331 CG321 CG321 SG3O1, penalty= 20.9

CG321 CG321 CG321 PG0 0.1100 3 0.00 ! lig , from CG331 CG321 CG321 SG3O1, penalty= 20.9

PG0 CG321 CG321 HGA2 0.0100 1 0.00 ! lig , from SG3O1 CG321 CG321 HGA2, penalty= 20

CG321 CG321 PG0 CG2R61 0.0500 3 0.00 ! lig , from CG2R61 CG321 PG1 OG2P1, penalty= 157.5

HGA2 CG321 PG0 CG2R61 0.0901 3 0.00 ! lig , from HGA3 CG331 PG0 OG2P1, penalty= 70

IMPROPERS

END
